# Endosomal protein DENND10/FAM45A integrates extracellular vesicle release with cancer cell migration

**DOI:** 10.1186/s12915-024-01948-4

**Published:** 2024-07-10

**Authors:** Shenqing Sun, Qian Li, Ganggang Liu, Xiaoheng Huang, Aiqing Li, Haoran Guo, Lijuan Qi, Jie Zhang, Jianrui Song, Xiong Su, Yanling Zhang

**Affiliations:** 1 School of Life Sciences, Suzhou Medical College of Soochow University, Suzhou, 215123 China; 2 Wisdom Lake Academy of Pharmacy, Jiangsu Provincial Higher Education Key Laboratory of Cell Therapy Nanoformulation, Xi’an Jiaotong-Liverpool University, Suzhou, 215123 China; 3 MOE Key Laboratory of Geriatric Diseases and Immunology, Suzhou Medical College of Soochow University, Suzhou, 215123 China; 4 Suzhou Key Laboratory of Systems Biomedicine, Suzhou Medical College of Soochow University, Suzhou, 215123 China

**Keywords:** DENND10, Extracellular vesicle, Migration, Breast cancer, Extracellular matrix

## Abstract

**Background:**

Mounting evidence shows that tumor-derived extracellular vesicles (EVs) are critical constituents in the tumor microenvironment. The composition and function of EVs often change during cancer progression. However, it remains less clear how cancer cells modulate their own EV biogenesis to promote tumor development. The release of EVs is closely linked to the endolysosome system residing within the cell. The current study aims to decipher the role of endosomal protein DENND10 in cancer development.

**Results:**

Bioinformatics data mining showed that DENND10 expression is significantly associated with poor prognosis in multiple cancer types and up-regulated in metastatic breast cancer cell lines. DENND10 knockout (DENND10-KO) in breast cancer cells led to defective EV biogenesis due to impaired endolysosomal trafficking. Intriguingly, DENND10-KO cells exhibited reductions in cell spreading, migration, invasion, and metastatic potential in vivo. These deficiencies in cell motility were associated with compromised cytoskeleton organization. Importantly, wild-type conditioned medium or EVs restored the migratory ability and cytoskeletal organization of DENND10-KO cells. Global proteomic profiling revealed that DENND10 depletion led to a distinct EV compositional landscape with remodeled profiles of extracellular matrix (ECM) and adhesion molecules. Consistently, exogenous application of ECM molecules rescued the spreading and migration of DENND10-KO cells.

**Conclusions:**

In summary, our study unveiled DENND10 as an intrinsic regulator of cell migration that modifies the tumor microenvironment through autocrine EV release, which could be exploited for developing targeted therapies for tumor metastasis.

**Supplementary Information:**

The online version contains supplementary material available at 10.1186/s12915-024-01948-4.

## Background

In recent years, extracellular vesicles (EVs), such as exosomes and microvesicles, have emerged as critical components of the tumor microenvironment [1]. EVs are membrane-enclosed particles secreted by all types of cells. They communicate with recipient cells in various ways, including binding to cognate surface receptors, forming adhesion hotspots, or direct internalization by target cells [2]. Diverse cell types within the tumor microenvironment generate EVs as autocrine or paracrine signals. These signals facilitate tumor migration and invasion, mediate immune suppression, enhance angiogenesis, and promote the formation of pre-metastatic niches [3, 4].

Multiple studies have revealed an association between EV composition and tumor development stages. For example, EVs from late-stage melanoma patients have higher protein concentrations and exhibit a greater capacity to stimulate metastasis than all other stages [5]. In another study, EVs secreted by KRAS mutant colon cancer cells display enhanced invasive capabilities compared to EVs from nontransformed cells [6]. Similarly, it was reported that serum EVs from patients with triple-negative breast cancers have a distinct proteomic landscape compared to EVs from healthy individuals [7]. Another study demonstrated that proteasome contents in EVs from patients with metastatic gastric cancer are significantly elevated compared to non-metastatic gastric cancer patients and healthy individuals [8]. These findings suggest a potential strategy for specifically targeting malignant tumors via modulating the release and composition of EVs. However, how cancer cells modulate their EV biogenesis to promote tumor progression remains little understood.

The biogenesis of EVs is closely linked to the homeostasis of the endolysosome system inside the cell [9, 10]. In searching for novel regulators of EV biogenesis, we identified an endosomal protein DENND10 (previously known as FAM45A or ANR3), which mainly localizes in MVEs/lysosomes and regulates the positioning and maturation of endosomes [11]. Knockdown of DENND10 in Hela cells reduced secretion of several classical EV markers [11], implying that it might modulate the biogenesis of EVs. DENND10 belongs to the family of DENN (differentially expressed in normal cells and neoplasia) domain-containing proteins, the largest family of guanine nucleotide exchange factors (GEFs) for Rab small GTPases. Human DENND10 protein shares 69.0% sequence similarity with its orthologue in amoeba (*Dictyostelium discoideum*) [11], suggesting conserved function(s) from unicellular organisms to vertebrates. Several recent structural studies have demonstrated that DENND10 is an integral subunit within the CCC/Retriever complexes (also called the Commander complex) in the endosomal recycling pathway [12–14]. However, no physiological roles for DENND10 have been described so far.

In the current study, we showed that DENND10 is important for the migration and invasion of breast cancer cells by regulating both the quantity and composition of EVs. DENND10 expression levels are associated with adverse prognosis of breast cancer patients. Suppression of DENND10 expression resulted in reduced secretion of EVs and impaired cell motility in breast cancer cells. Notably, the application of wild-type cells-derived EVs rescued the migration defects of DENND10 knockout cells. Furthermore, we showed that DENND10 knockout EVs had a distinct proteomic composition, particularly in ECM and adhesion molecules. Accordingly, exogenously added ECMs rescued the DENND10 knockout phenotypes. These results revealed a novel intrinsic mechanism in tumor cells that modulates their microenvironment by autocrine EV release, which could be utilized for designing new strategies to suppress metastasis.

## Results

### DENND10 expression is associated with the prognosis of breast cancer patients

**Fig. 1:**
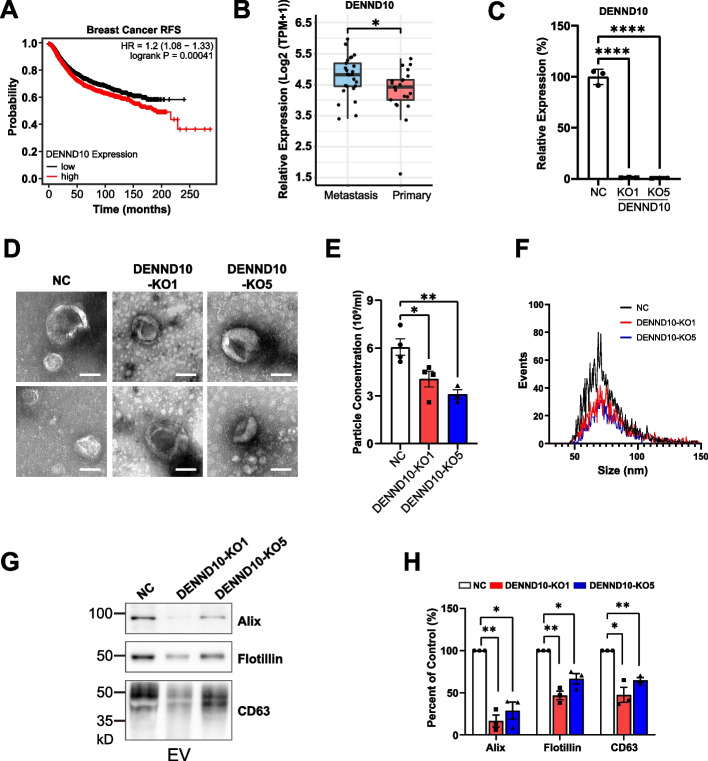
DENND10 is important for EV biogenesis in breast cancer cells. **A** High *DENND10* expression is associated with poor relapse-free survival (RFS) in breast cancer patients. Patients were divided into high expression (red) and low expression (black) based on the median expression of *DENND10*. *N* = 4, 929. HR, hazard ratio. Data source: KM Plotter. **B** *DENND10* expression is higher in adherent human breast cancer cell lines derived from metastatic sites than those primary sites. Data source: Cancer Cell Line Encyclopedia (CCLE). **C** Establishment of DENND10 knockout cells. 4T1 cells were infected with lentivirus encoding CRISPR guide RNAs targeting the *Dennd10* gene. Two single clones were selected, DENND10-KO1 and DENND10-KO5, that had near-zero expression of Dennd10 shown by qRT-PCR. Error bars, SEM (*N* = 3, see Additional file 3). Significance was determined by one-way ANOVA followed by Dunnett’s multiple comparisons test. ****, *p* < 0.0001. **D** Morphology of EVs isolated from NC and DENND10-KO cells was imaged by TEM. **E–F** Particle concentration (**E**) and size distribution (**F**) of EVs were measured by NTA. EVs were isolated from conditioned medium collected from three 10-cm dishes and resuspended in 200 µl PBS. Error bars, SEM (*N* = 3–4, see Additional file 3). Significance in (**E**) was determined by one-way ANOVA followed by Dunnett’s multiple comparisons test. *, *p* < 0.05; **, *p* < 0.01. **G** Protein levels of EV markers, including Alix, Flotillin-1, and CD63, in EVs derived from equal numbers of NC and DENND-KO cells. **H** Quantitation of protein levels in (**G**). Error bars, SEM (*N* = 3, see Additional file 3). Significance was determined by Student’s t-test with Welch correction. *, *p* < 0.05; **, *p* < 0.01

Previously, we found that DENND10/FAM45A knockdown leads to reduced secretion of EV proteins in Hela cells [11]. As EVs are critical components of tumor microenvironment, we wondered whether DENND10 is involved in the progression of any type of cancer. First, we analyzed the association between DENND10 expression and the survival of cancer patients with KM Plotter [15], a web-based portal that integrates clinical data from multiple sources, including GEO, EGA, and TCGA. Intriguingly, high expression of *DENND10* was significantly correlated with poor relapse-free survival (*p* = 0.00041) in breast cancer patients (Fig. 1A). When these patients were stratified into major molecular subtypes [16], this trend was evident across the subtypes of basal, luminal A, and luminal B patient cohorts at various significance levels (Additional file 1: Figure S1A). The most significant impact (*p* = 0.00043) was observed in the luminal B subtype (Additional file 1: Figure S1A), which is characterized by high levels of the proliferative marker Ki67 and generally worse prognosis. This finding is consistent with a role for DENND10 in progressive cancer subtypes. A similar correlation was also observed in gastric cancer patients, where high *DENND10* expression was significantly associated with poor overall survival (*p* = 0.006) and a short duration of time to first-progression (*p* = 0.0018) (Additional file 1: Figure S1B). In addition, analysis of the CCLE (Cancer Cell Line Encyclopedia) database [17] revealed that *DENND10* expression was higher in human breast cancer cell lines derived from metastatic sites compared to those from primary sites (Fig. 1B, Additional file 2: Table S1), strongly suggesting that DENND10 might be involved in breast cancer metastasis. Consistent with this notion, high DENND10 expression was correlated with shorter distant-metastasis free survival in a public breast cancer dataset (GSE46563) [18, 19] (Additional file 1: Figure S1C). These results suggested that *DENND10* plays an important role in cancer progression.

### DENND10 is important for EV biogenesis in breast cancer cells

To decipher the role of DENND10 in breast cancer cells, we knocked out the *Dennd10* gene in 4T1 cells, a highly metastatic triple-negative breast cancer cell line, using lentivirus-based CRISPR/Cas9 gene editing. Two independent single clone-derived knockout lines, DENND10-KO1 and DENND10-KO5, were utilized in subsequent experiments to account for clonal heterogeneity. *Dennd10* mRNA levels were close to zero in both knockout cell lines (Fig. 1C). Proliferation rates of DENND10-KO cells did not significantly differ from cells infected with a non-targeting control vector (NC) (Additional file 1: Figure S2), suggesting that DENND10 is not required for the proliferation of 4T1 cells.

Next, we examined the effect of DENND10 knockout on EV biogenesis in 4T1 cells. EVs were isolated from the conditioned medium of NC and DENND10-KO cells. Transmission electron microscopy (TEM) imaging revealed that NC and DENND10-KO EVs were morphologically similar and exhibited a classical cup-shaped appearance (Fig. 1D). However, the number of EVs in DENND10-KO cells, determined by nano flow cytometry (nFCM), decreased to 51–67% of NC cells (Fig. 1E), although the distribution of puncta diameter did not change (Fig. 1F). Furthermore, we measured the levels of EV markers, including Alix, Flotillin-1, and CD63, in EV lysates derived from an equal number of cells (Fig. 1G-H). All three markers were consistently decreased in EV lysates derived from DENND10-KO cells, suggesting impaired EV biogenesis.

**Fig. 2:**
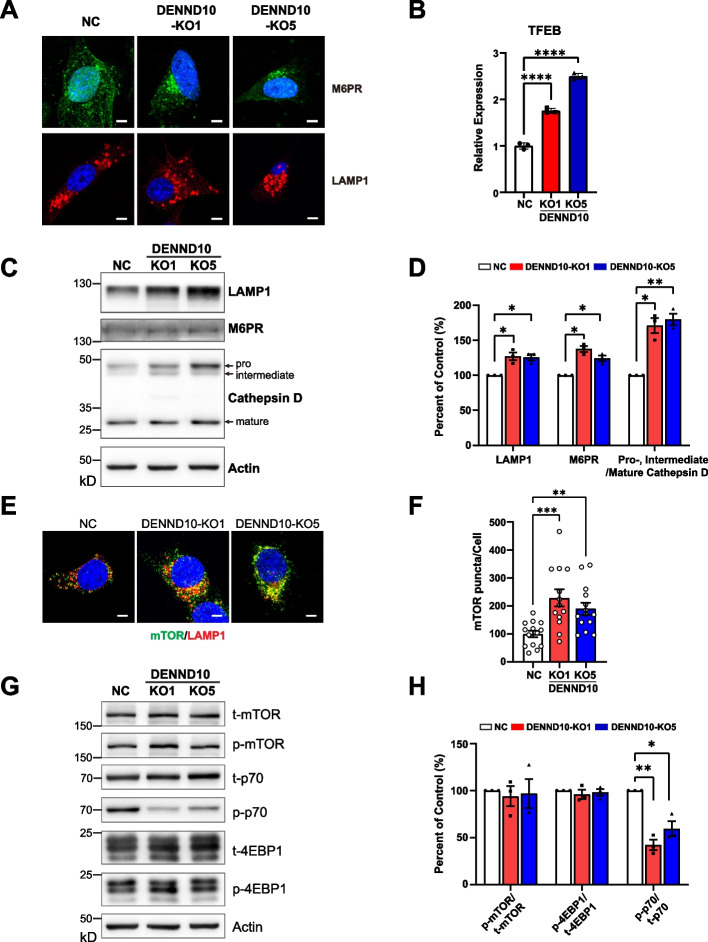
DENND10 deletion in 4T1 cells leads to perturbed endolysosomal homeostasis and partial defects in the mTOR pathway. **A** Distribution of M6PR, which cycles between trans-Golgi network and endosomes, and LAMP1, which marks late endosomes/lysosomes, in NC and DENND10-KO cells. **B** Expression levels of TFEB mRNA in NC and DENND10-KO cells. Error bars, SEM (*N* = 3, see Additional file 3). Significance was determined with Student’s t-test. ****, *p* < 0.0001. **C** Protein levels of LAMP1, M6PR, and Cathepsin D in NC and DENND10-KO cells. The pro-, intermediate, and mature forms of Cathepsin D were shown. **D** Quantitation of protein levels in (**C**). SEM (*N* = 3, see Additional file 3). Significance was determined by Student’s t-test with Welch correction. *, *p* < 0.05; **, *p* < 0.01. **E** Distribution of mTOR (green) and LAMP1 (red) in NC and DENND10-KO cells. **F** Quantitation of the number of mTOR puncta per cell in NC or DENND10-KO. *N* = 13–14 cells from three independent experiments. Significance was determined with the Kruskal–Wallis test, followed by Dunn’s multiple-comparison test. **, *p* < 0.01; ***, *p* < 0.001. **G** Expression levels of total mTOR (t-mTOR), phosphorylated mTOR (p-mTOR), total p70-S6K (t-p70), phosphorylated p70-S6K (p-p70), total 4EBP1 (t-4EBP1), and phosphorylated 4EBP1 (p-4EBP1) were detected by Western blotting. **H** Quantitation of protein levels in (**G**). The mTOR pathway was probed by the ratio between phosphorylated mTOR, 4EBP1, p70, and corresponding total proteins. All values were normalized to NC cells. Error bars, SEM (*N* = 3, see Additional file 3). Significance was determined by Student’s t-test with Welch correction. *, *p* < 0.05; **, *p* < 0.01

Given the endosomal localization of DENND10 protein [11], this reduction in EV biogenesis is likely a result of dysregulated endomembrane system. In order to determine the effect of DENND10 deletion on the endolysosome system in breast cancer cells, we examined the distribution of several endocytic markers, including mannose-6-phosphate receptor (M6PR), a sorting receptor for lysosomal enzymes, and LAMP1, an integral membrane protein on late endosomes/lysosomes. We found that M6PR vesicles were scattered in the cytoplasm in NC cells, but markedly clustered in the perinuclear region in DENND10-KO cells (Fig. 2A, Additional file 1: Figure S3A-B). LAMP1 was also clustered in DENND10-KO cells, though less prominent (Fig. 2A, Additional file 1: Figure S3A-B). TFEB, a master transcription factor for lysosome biogenesis, was strongly up-regulated in DENND10-KO cells (239%—324%, Fig. 2B), a phenomenon frequently found in cells with lysosomal stress [20]. Consistently, immature precursors of Cathepsin D, a lysosomal protease, were accumulated in DENND10-KO cells (Fig. 2C-D), suggesting delayed trafficking to lysosomes. Furthermore, protein levels of both LAMP1 and M6PR were elevated in DENND10-KO cells (Fig. 2C-D), which could be attributed to the compensatory increase of lysosomal biogenesis. Autophagy levels were marginally higher in DENND10-KO cells, but it was not statistically significant (Additional file 1: Figure S3C-D). We assessed lysosomal protease activities using the Cathepsin B MR (Magic Red) substrate, which becomes fluorescent upon cleavage by Cathepsin B enzymes inside lysosomes. Remarkably, the intensity of the MR puncta was significantly decreased in DENND10-KO cells, suggesting reduced proteolytic ability within individual lysosomes (Additional file 1: Figure S3E-F).

Since lysosomes are sites of activation for the mTORC1 kinase complex [21], we also examined the mTORC1 pathway. Consistent with previous literature [22], mTOR colocalized with LAMP1-labeled lysosomes (Fig. 2E). The number of mTOR puncta was significantly increased in DENND10-KO cells (Fig. 2F). DENND10 depletion did not alter the phosphorylation of mTOR and one of its substrates, 4EBP1; however, phosphorylation of another mTOR substrate, p70-S6K, was reduced in DENND10-KO cells (Fig. 2G-H), suggesting partial defects in mTOR signaling. Overall, these results demonstrated that loss of DENND10 resulted in impaired traffic in the endolysosome system in 4T1 cells, which could eventually lead to reduced EV biogenesis.

### DENND10 regulates cancer cell spreading and migration via EVs

**Fig. 3:**
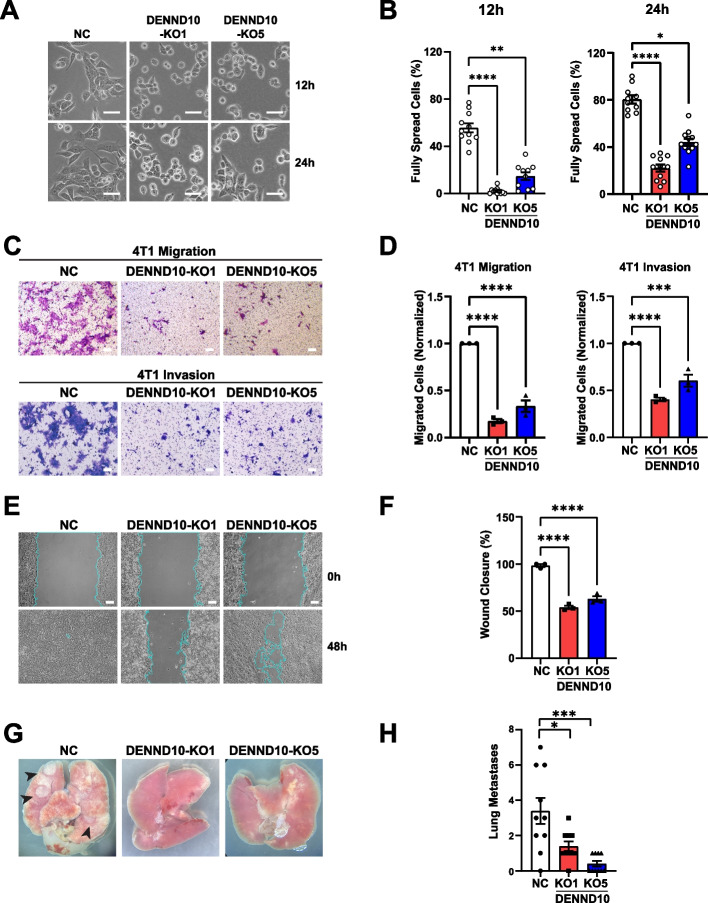
DENND10 is involved in cell spreading, migration, and invasion. **A** DENND10 deletion results in impaired cell spreading. NC and DENND10-KO cell lines were imaged at 12 and 24 h after plating. **B** Quantitation of cell spreading at 12 and 24 h after plating. The percentage of fully spread cells was measured for each field. Error bars, SEM. *N* = 10–12 random fields from three independent experiments. Significance was determined with the Kruskal–Wallis test, followed by Dunn’s multiple-comparison test. *, *p* < 0.05; **, *p* < 0.01; ****, *p* < 0.0001. **C** DENND10 deletion leads to defective cell migration and invasion across the Transwell membrane. Top: migration. Bottom: invasion through Matrigel. Scale bars: 100 μm. **D** Quantitation of migrated/invaded cells in (**C**). The number of migrated/invaded cells per field was counted and normalized to the corresponding control. Error bars, SEM (*N* = 3, see Additional file 3). Significance was determined by one-way ANOVA followed by Dunnett’s multiple comparisons test. ****, *p* < 0.0001. **E** DENND10 deletion leads to defective wound closure in the scratch assay. Confluent NC and DENND10-KO 4T1 cell cultures were scratched and allowed to migrate for 48 h in serum-free medium. The wound area is marked with a blue outline. Scale bar: 100 μm. **F** Quantitation of the percentage of closed wound area in (**E**). Error bars, SEM (*N* = 3, see Additional file 3). Significance was determined by one-way ANOVA followed by Dunnett’s multiple comparisons test. ****, *p* < 0.0001. **G-H** DENND10-KO and NC 4T1 cells were inoculated subcutaneously in the mammary pad of BALB/c mice. After four weeks, macro-metastases of DENND10-KO and NC cells to the lung were manually counted. Error bars, SEM (*N* = 10 animals per group). Significance was determined with the Kruskal–Wallis test, followed by multiple-comparison correction. ns, non-significant; *, *p* < 0.05; ***, *p* < 0.001

During routine passaging, DENND10-KO cells were noticeably more round-shaped, implying decreased adherence to the substratum and/or inability to protrude the plasma membrane. More than half of NC cells developed a flattened morphology at 12 h after plating, and ~ 80% of NC cells completed spreading at 24 h (Fig. 3A-B). In contrast, only ~ 2% of DENND10-KO1 and ~ 15% of DENND10-KO5 cells achieved flattened morphology at 12 h, and less than half spread out at 24 h (Fig. 3A-B). This spreading defect did not lead to an increase in detachment-related cell death (i.e., anoikis) (Additional file 1: Figure S4A-B). Since cell-ECM adhesion and membrane remodeling play critical roles in cell migration [23], we assessed the motility of DENND10-KO cells. The numbers of DENND10-KO 4T1 cells that migrated through Transwell membrane pores or invaded through Matrigel coating were significantly fewer than NC cells (Fig. 3C-D). Wound scratch assay showed that, while NC cells almost entirely closed the wound within 48 h, a significant wound gap remained in DENND10-KO cells (Fig. 3E-F). No changes in cell viability were observed in DENND10-KO cells during the same time frame (Additional file 1: Figure S4C-D). We also determined the effect of DENND10 deletion on cancer metastasis in vivo. NC and DENND10-KO 4T1 cells were injected into BALB/c mice and examined after four weeks. Notably, the number of macro-metastases to the lung was significantly lower for both DENND10-KO cell lines (Fig. 3G-H), indicating that loss of DENND10 function decreased the metastasis potential of 4T1 cells in vivo. Additionally, we knocked down *DENND10* expression in another metastatic triple-negative breast cancer cell line, MDA-MB-231. A similar reduction in migrated cells was observed with two independent short hairpin RNA (shRNA) constructs against *DENND10* (Additional file 1: Figure S5A-B). Taken together, these results indicated that DENND10 is important for the migration potential of breast cancer cells.

**Fig. 4:**
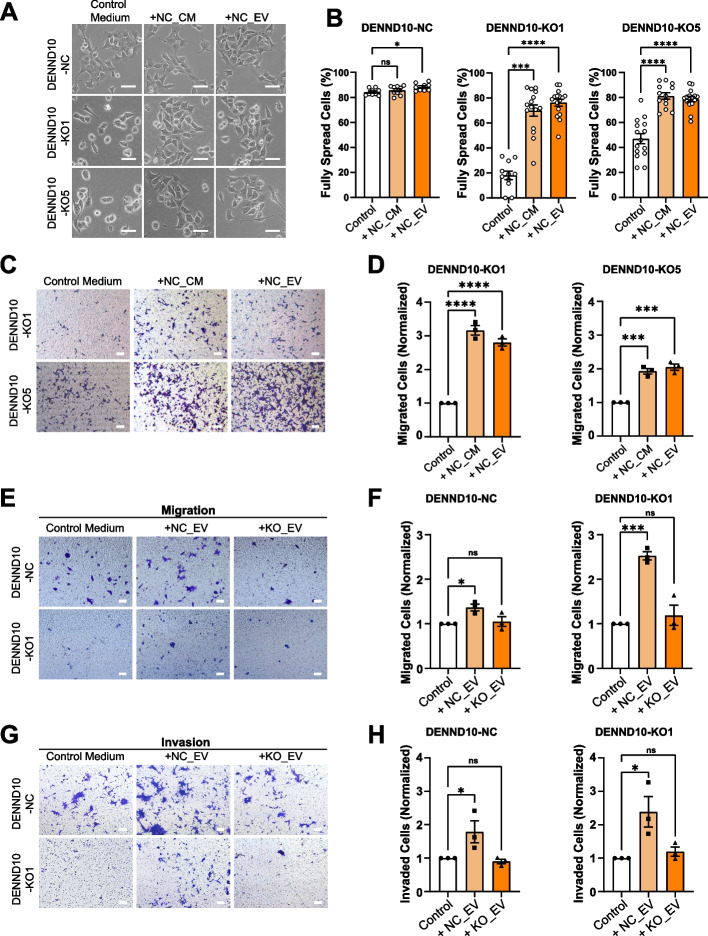
Wild-type EVs rescue the impaired cell spreading and migration in DENND10-KO cells. **A** NC and DENND10-KO cells were cultured in control medium, or medium supplemented with 62.5% NC conditional medium or 10 µg/ml NC EVs. Cell morphology was imaged at 24 h after plating. **B** Quantitation of cell spreading in (**A**). Error bars, SEM. *N* = 9–16 random fields from three independent experiments. Significance was determined with the Kruskal–Wallis test, followed by Dunn’s multiple-comparison test. ns, non-significant; *, *p* < 0.05; ***, *p* < 0.001; ****, *p* < 0.0001. **C** NC-derived conditioned medium or EVs rescued the migration of DENND10-KO cells. DENND10-KO cells were seeded at 1 × 10^5^ cells/well in control serum-free medium, or medium supplemented with 62.5% serum-free NC conditioned medium or 10 µg/ml NC EVs. Cells that migrated across the membrane were imaged. Scale bars: 100 μm. **D** Quantitation of cell migration in (**C**). Error bars, SEM (*N* = 3, see Additional file 3). Statistical significance was analyzed by one-way ANOVA followed by Dunnett’s multiple comparisons test. ***, *p* < 0.001; ****, *p* < 0.0001. **E–H** NC-derived EVs, but not DENND10-KO-derived EVs of equal mass, rescued the migration (**E**) and invasion (**G**) of DENND10-KO cells. NC and DENND10-KO cells were seeded at 5 × 10^4^ cells/well in control serum-free medium, or 10 µg/ml NC EVs, or 10 µg/ml KO EVs. Scale bars: 100 μm. (**F)** and (**H**) are quantitation of cells that moved across the Transwell membrane in (**E**) and (**G**), respectively. Error bars, SEM (*N* = 3, see Additional file 3). Statistical significance in (**F**) and (**H**) was analyzed as in (**D**). ns, non-significant; *, *p* < 0.05; ***, *p* < 0.001

We next sought to understand the mechanism by which DENND10 regulates cancer cell migration. We noticed that better spreading of DENND10-KO cells could be achieved by longer culturing time and higher cell density, suggesting a secretion defect. Therefore, we investigated whether defects in DENND10-KO cells could be rescued by additional exogenous EVs. Firstly, DENND10-KO cells were cultured in the presence of NC-derived conditioned medium (NC_CM) or EVs purified from NC-conditioned medium (NC_EVs). Significantly, the spreading of DENND10-KO cells was increased to ~ 70% to 81% at 24 h after treating with NC_CM or NC_EVs (Fig. 4A-B), which strongly suggests that defective cell spreading in DENND10-KO cells is mainly due to impaired EV secretion. Next, we treated DENND10-KO cells with NC_CM or NC_EVs during Transwell migration. Both treatments significantly increased the number of migrated DENND10-KO cells across the membrane filter (Fig. 4C-D). Lastly, we compared the stimulatory efficiency of EVs derived from NC or DENND10-KO cells. With equal masses (10 µg/ml) of co-incubated EVs, NC-derived EVs, but not DENND10-KO-derived EVs, could significantly increase the migration (Fig. 4E-F) and invasion (Fig. 4G-H) of DENND10-KO cells, suggesting that DENND10-KO_EVs contained fewer amounts of cargoes that stimulate cell motility. Together, these results indicate that DENND10 regulates cell spreading and migration by modulating autocrine EV release.

### Loss of DENND10 results in impaired cytoskeleton organization

**Fig. 5:**
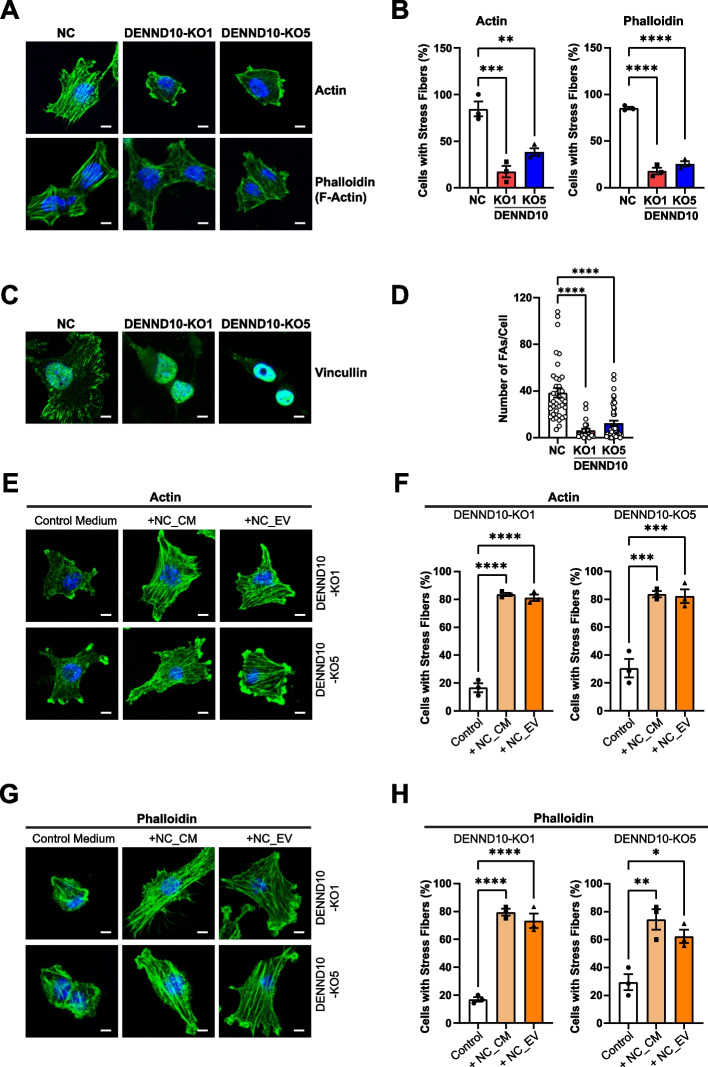
Loss of DENND10 results in cytoskeleton reorganization. **A** NC and DENND10-KO cells were cultured for 24 h before fixation and stained with anti-actin and phalloidin. Scale bars, 5 μm. **B** Quantitation of the percentage of cells with stress fibers in (**A**). Error bars, SEM (*N* = 3, see Additional file 3). Statistical significance was analyzed by one-way ANOVA followed by Dunnett’s multiple comparisons test. **, *p* < 0.001; ***, *p* < 0.001; ****, *p* < 0.0001. **C** Focal adhesions in NC and DENND10-KO cells were visualized by anti-vinculin staining. Scale bars, 5 μm. **D** Quantitation of the number of focal adhesions (FAs) per cell in (**C**). Error bars, SEM. *N* = 23–43 cells from three independent experiments. Significance was determined with the Kruskal–Wallis test, followed by Dunn’s multiple-comparison test. ****, *p* < 0.0001. **E–H** DENND10-KO cells were cultured in control medium, or treated with NC-derived conditioned medium or 10 µg/ml EVs for 24 h, and then stained with anti-actin (**E**) or phalloidin (**G**). Scale bars, 5 μm. (**F**) and (**H**) are quantitation of the percentage of cells with stress fibers in (**E**) and (**G**), respectively. Error bars, SEM (*N* = 3, see Additional file 3). Statistical significance was analyzed as in (**B**)

Subsequently, we determined the mechanism of cell motility defects in DENND10-KO cells. At the subcellular level, both cell spreading and migration involve a series of coordinated events that remodel cytoskeleton and membrane structures, including the formation of stress fibers, focal adhesions, and membrane ruffling [24, 25]. In order to determine whether DENND10 participates in these events, we first examined cytoskeleton organization in NC and DENND10-KO cells. In NC cells, both anti-actin immunofluorescence and filamentous actin (F-actin) staining by phalloidin showed strong parallel stress fibers across the cell body (Fig. 5A-B). In contrast, in DENND10-KO cells, only the cortical region was stained strongly for actin, with no prominent stress fibers present in the cell interior (Fig. 5A-B). Total levels of actin proteins were not changed (Additional file 1: Figure S6A). At the same time, the microtubule network stained by anti-tubulin also appeared disorganized in DENND10-KO cells (Additional file 1: Figure S7A-B). Subsequently, we determined whether focal adhesions were affected by DENND10 deletion with anti-vinculin immunostaining. Focal adhesions are complex structures that connect the cytoskeleton to ECM through integrin receptors. We found that focal adhesions were remarkably diminished in DENND10-KO cells (Fig. 5C-D), consistent with weak adherence between cells and the ECM. These results indicated global changes in cytoskeletal organization in DENND10-KO cells and, in particular, reduction of stress fibers and focal adhesions.

Next, we treated cells with the phorbol ester PMA, a PKC agonist that promotes membrane ruffling and cell spreading [26]. After PMA exposure, multiple actin-rich membrane ruffles appeared in both NC and DENND10-KO cells (arrowheads in Additional file 1: Figure S7C). However, stress fibers still failed to form in PMA-treated DENND10-KO cells (Additional file 1: Figure S7C-D), suggesting membrane ruffling was not responsible for defective cell spreading in DENND10-KO cells.

Subsequently, we examined the effect of NC_CM or NC_EV on cytoskeleton organization in DENND10-KO cells. Both treatments induced strong stress fiber formation in DENND10-KO cells, as shown by anti-actin and phalloidin staining (Fig. 5E-H), indicating that cargoes carried in secreted EVs could rectify intracellular organization of the cytoskeleton network. Collectively, the results above indicated that DENND10 regulates the assembly of adhesion structures and cell motility via autocrine EVs.

### DENND10 deletion leads to altered proteomic composition of EVs

**Fig. 6:**
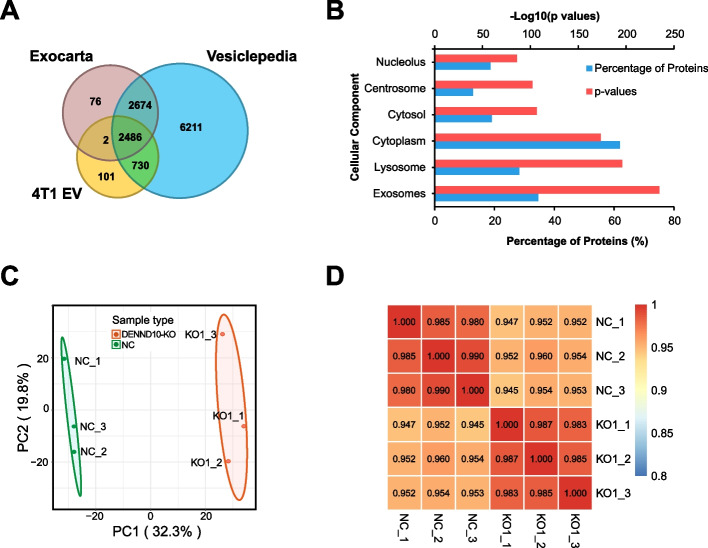
Overall proteome landscape of EVs derived from NC and DENND10-KO cells. **A** Venn diagram comparing proteins identified in 4T1 EVs (including both NC and DENND10-KO) and known EV proteins deposited in Vesiclepedia and Exocarta. Gene IDs were used for comparison. Four proteins in the 4T1-EV dataset were not included due to unmappable or redundant IDs. **B** Enrichment analysis of cellular components in identified 4T1 EV proteins calculated by FunRich. **C** Principal component analysis (PCA) of all EV samples. The percentages of variation accounted for by PC1 and PC2 were indicated. The biological factor (NC vs DENND10-KO) was highly correlated with PC1. **D** Pearson correlation coefficients among all EV samples. Similarities within and between groups were clearly distinguished

As the biological activities of EVs depend on specific cargoes they carry, we sought to ask whether DENND10 depletion influenced the proteomic composition of EVs. Four-dimensional quantitative mass spectrometry [27] was utilized to profile EV samples isolated from NC and DENND10-KO1 conditioned medium [28]. Three biological replicates were employed for each cell line to allow statistical comparison. A total of 3323 proteins were identified, among which 2518 proteins were quantifiable in both cell lines (Additional file 2: Table S2). Notably, DENND10 proteins were present in NC EVs and, as expected, undetectable in all DENND10-KO EVs (Additional file 2: Table S3), further validating the knockout efficiency at the protein level. When compared to two public databases of EV cargoes, Vesiclepedia [29] and Exocarta [30], 3216 (> 95%) proteins in our dataset overlapped with Vesiclepedia, and 2486 (~ 75%) proteins overlapped with both databases simultaneously (Fig. 6A, Additional file 2: Table S4). In addition, cell component (CC) analysis revealed that “exosomes” was the most significantly enriched term (adjusted *p* = 1.5e-235) among all identified proteins (Fig. 6B, Additional file 2: Table S5), again confirming that these proteins were mainly derived from EVs. Of importance, principal component analysis (PCA) showed that NC and DENND10-KO EVs were well separated by the first principal component (PC1) alone (Fig. 6C). Pearson correlation also grouped these two types of EVs into separate clusters despite high similarity (Pearson correlation > 0.9) among all samples (Fig. 6D), indicating that loss of DENND10 resulted in an altered proteomic EV profile.

**Fig. 7:**
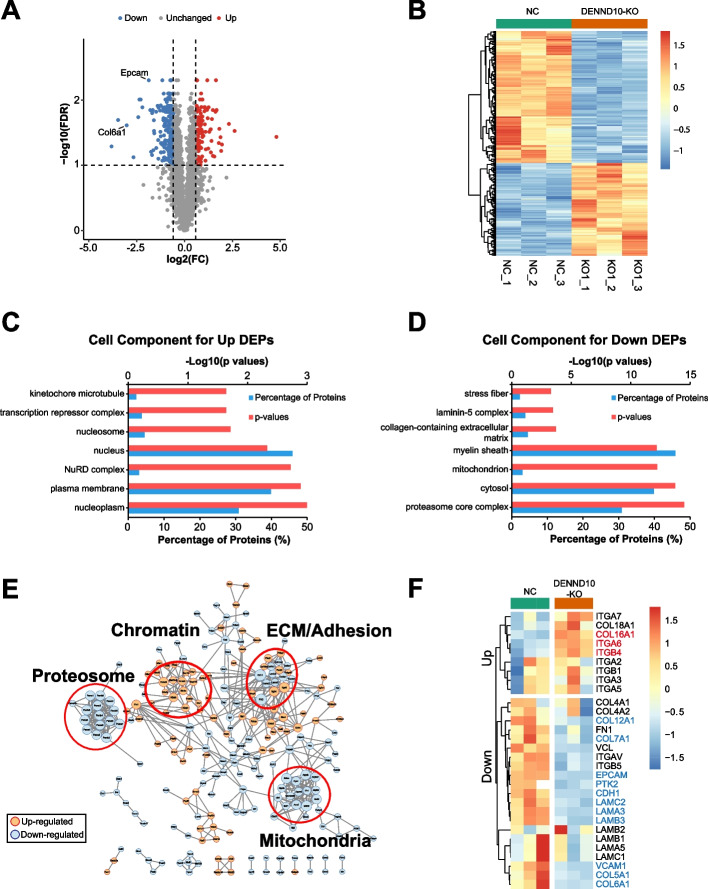
DENND10 deletion results in altered EV composition. **A** Volcano plot showing –log_10_(FDR) and log_2_(FC) of quantifiable proteins. Each dot represents one protein. Thresholds for defining DEPs were portrayed as dashed lines. Up-regulated DEPs were colored red, and down-regulated DEPs were colored blue. **B** Heatmap of DEPs showing the expression of each DEP in all samples. Expression values were centered to the mean and normalized to the variance across all samples. **C-D** Enrichment analysis of cellular components in up-regulated (**C**) and down-regulated (**D**) DEPs, calculated by FunRich. **E** Protein–protein interaction network among all DEPs derived from the STRING database. Up-regulated DEPs were colored light red, and down-regulated DEPs were colored light blue. Node size was correlated with the number of degrees of each node. Major clusters of proteins were circled and labeled. **F** Heatmap of selected proteins involved in cell adhesion and ECMs, including integrin subtypes, focal adhesion proteins (Vinculin/VCL, FAK/PTK2), ECM proteins (fibronectin, collagen, laminin), and cell–cell adhesion proteins (EPCAM). The names of up-regulated and down-regulated DEPs were highlighted in red and blue, respectively. COL1A1 was not detected in DENND10_KO EVs (Additional file 2: Table S3) and therefore not displayed here

Next, differentially expressed proteins (DEPs) between NC and DENND10-KO EVs were determined using Student’s t-test followed by correction for multiple comparisons. DEPs were defined as fold changes (FC) greater than 1.5, *p* < 0.01, and false discovery rate (FDR) < 0.1. A total of 135 up-regulated and 196 down-regulated proteins were obtained (Fig. 7A-B, Additional file 2: Table S6). These DEPs corresponded to over-represented and under-represented proteins in DENND10-KO EVs, respectively, since equal masses of EV samples were used for analysis. Notably, collagen molecule COL6A1 and epithelial cell adhesion protein EPCAM were among the top down-regulated DEPs (Fig. 7A), consistent with reduced adherence in DENND10-KO cells. ECMs represent a crucial category of EV cargoes consistently identified across multiple proteomics studies [31–33]. They are located on the surface of EVs [34, 35] and could form adhesion hotspots to guide cell migration [36–39]. Note that commonly used EV markers, including Alix/Pdcd6ip, CD63, CD9, Tsg101, Flotillin, Syntenin-1, and CD81, were all detected in our dataset, and did not significantly differ between NC and DENND10-KO EVs of equal masses (Additional file 2: Table S3). This aligns with our observation that DENND10-KO did not significantly alter EV morphology and size distribution (Fig. 1 D and F).

To understand the biological implications of compositional changes of DENND10-KO EVs, cell component enrichment analysis was performed on DEPs. In the up-regulated DEPs, top component terms were mainly related to the nucleus and chromatin organization (Fig. 7C, Additional file 2: Table S7). In the down-regulated DEPs, top component terms had several distinct categories, including stress fiber-related proteins, ECM proteins (collagen and laminin), mitochondria, and proteasomes (Fig. 7D, Additional file 2: Table S7). In addition, we further visualized protein–protein interactions among all DEPs using the STRING database [40] (Fig. 7E, Additional file 2: Table S8). Several major clusters could be recognized in the interaction network of DEPs (Additional file 1: Figure S8). Firstly, a number of chromatin-binding proteins were up-regulated in DENND10-KO EVs (Additional file 1: Figure S8A). These included histone variant H2AX, which is associated with DNA damage response and increased cytoplasmic chromatin fragment [41]. Secondly, two major clusters of proteins were down-regulated in DENND10-KO EVs: mitochondrial enzymes, such as proteins involved in tricarboxylic acid cycle and electron transport chain (Additional file 1: Figure S8B), and proteasome-related proteins, including 20S proteasome subunits PSMA1/2/3/4/5/6/7 and PSMB1/2/3/4/5/6/8 (Additional file 1: Figure S8C). Finally, selected cell adhesion and ECM proteins were differentially altered, present in both up- and down-regulated DEPs, suggesting remodeled ECM profiles in DENND10-KO EVs (Additional file 1: Figure S8D).

We further examined EV-associated cell adhesion and ECM proteins (Fig. 7F, Additional file 2: Table S3), since these could directly contribute to cell motility. Firstly, integrin proteins, which mediate cell-ECM adhesion, were altered in subtype profiles in DENND10-KO EVs. Intriguingly, integrin α6 and β4 were up-regulated in DENND10-KO EVs, whereas integrin αV (FC = 0.687, FDR = 0.027) and β5 (FC = 0.677, FDR = 0.03) were moderately down-regulated, implying a possible “type-switching” for integrin proteins. Integrin β1 and β5, the canonical fibronectin receptor, did not change significantly. In addition, consistent with our earlier results on focal adhesions (Fig. 5C), focal adhesion associated proteins PTK2 (FAK) (FC = 0.497, FDR = 0.01) and VCL (Vinculin) (FC = 0.678, FDR = 0.01) were trended downwards in DENND10-KO EVs. Secondly, ECM constituents also exhibited some form of type switching. Fibrillar collagens (COL1A1, COL5A1), microfibril forming collagen (COL6A1), and anchoring fibril collagen (COL7A1) were down-regulated, whereas non-fibrillar FACIT family collagens COL16A1 were up-regulated (Fig. 7F, Additional file 2: Table S3). These changes would likely lead to alterations in the architecture and mechanical properties of collagen fibers. At least three laminin proteins (LAMA3, LAMB3, LAMC2) were significantly decreased in DENND10-KO EVs. Fibronectin (FN1) was moderately down-regulated (FC = 0.642, FDR = 0.107) but did not reach our DEP threshold. Finally, several cell–cell adhesion proteins, including EPCAM, CDH1, and VCAM1, were significantly decreased in DENND10-KO EVs, which could also contribute to the migration defects in DENND10-KO cells. A number of studies in recent years demonstrated that cell–cell adhesion proteins promote collective cell migration and facilitate metastasis in breast cancer patients [42–44]. Altogether, these results revealed global remodeling in cell adhesion and ECM composition in DENND10-KO EVs.

**Fig. 8:**
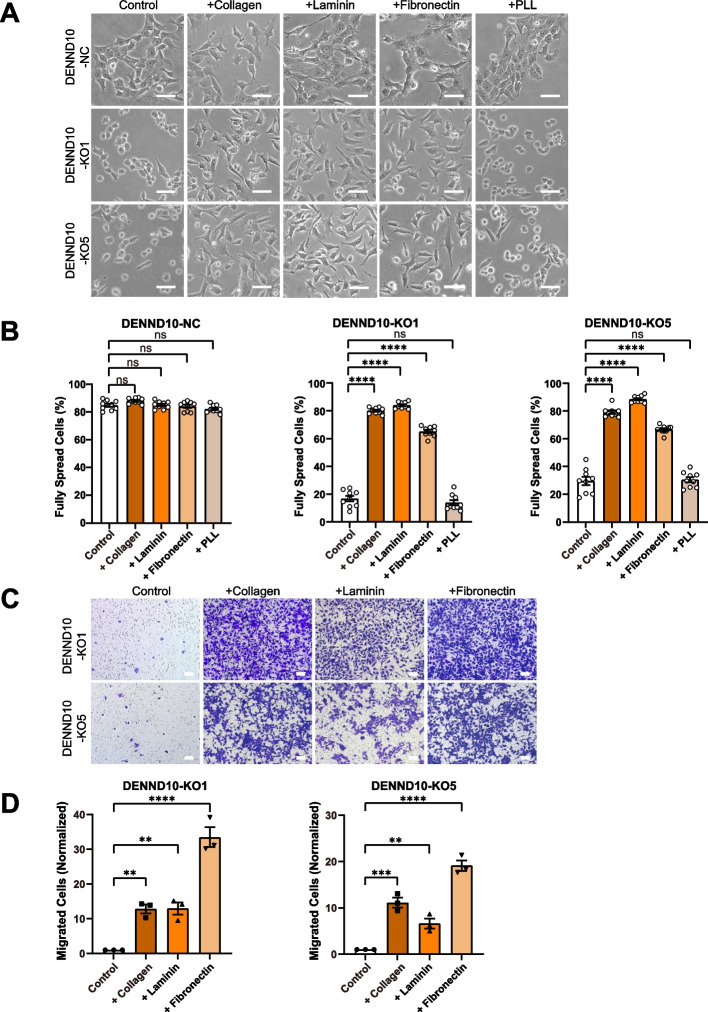
ECM supplementation rescues DENND10-KO 4T1 morphology and migration. **A** DENND10-NC and DENND10-KO cells were cultured on control substratum, or substratum coated with 15 µg/ml collagen I, laminin, fibronectin, or 0.1 mg/ml poly-L-lysine (PLL). Cell morphology was imaged at 24 h after plating. Scale bars: 25 μm. **B** Quantitation of cell spreading in (**A**). Error bars, SEM. *N* = 9 random fields from three independent experiments. Significance was determined with one-way ANOVA followed by Dunnett’s multiple-comparison test. ****, *p* < 0.0001, ns, non-significant. **C** Migration of DENND10-KO cells across ECM-coated filters was measured by the Transwell assay. Transwell filters were coated on the underside with 15 µg/ml collagen I, laminin, or fibronectin for 2 h before use. Cells that migrated across the membrane were imaged and normalized to the control. Scale bars: 100 μm. **D** Quantitation of cell migration in (**C**). Error bars, SEM (*N* = 3, see Additional file 3). Statistical significance was analyzed by one-way ANOVA followed by Dunnett’s multiple-comparison test. **, *p* < 0.01; ***, *p* < 0.001; ****, *p* < 0.0001

Considering that DENND10-KO cells had reduced stress fibers/focal adhesions and altered EV-associated ECM profiles, we examined whether exogenous supplementation of ECM proteins could rescue DENND10-KO phenotypes. Intriguingly, pre-coating cell dishes with collagen I, laminin, or fibronectin, rescued the spreading of DENND10-KO cells (Fig. 8A-B). At the same time, poly-L-lysine coating, which enhances electrostatic adhesion but does not engage integrin receptors, had no effect, demonstrating the specificity of this rescue. The migration of DENND10-KO cells across Transwell filters was also greatly enhanced by collagen, laminin, and fibronectin coating (Fig. 8C-D). These observations expanded the results of several previous studies [37, 38, 45], where EV-associated fibronectin deposition on the substratum was shown to be critical for cell migration. Together, our study demonstrated the importance of EV-associated ECM profiles in adhesion and migration and their regulation by DENND10.

**Fig. 9:**
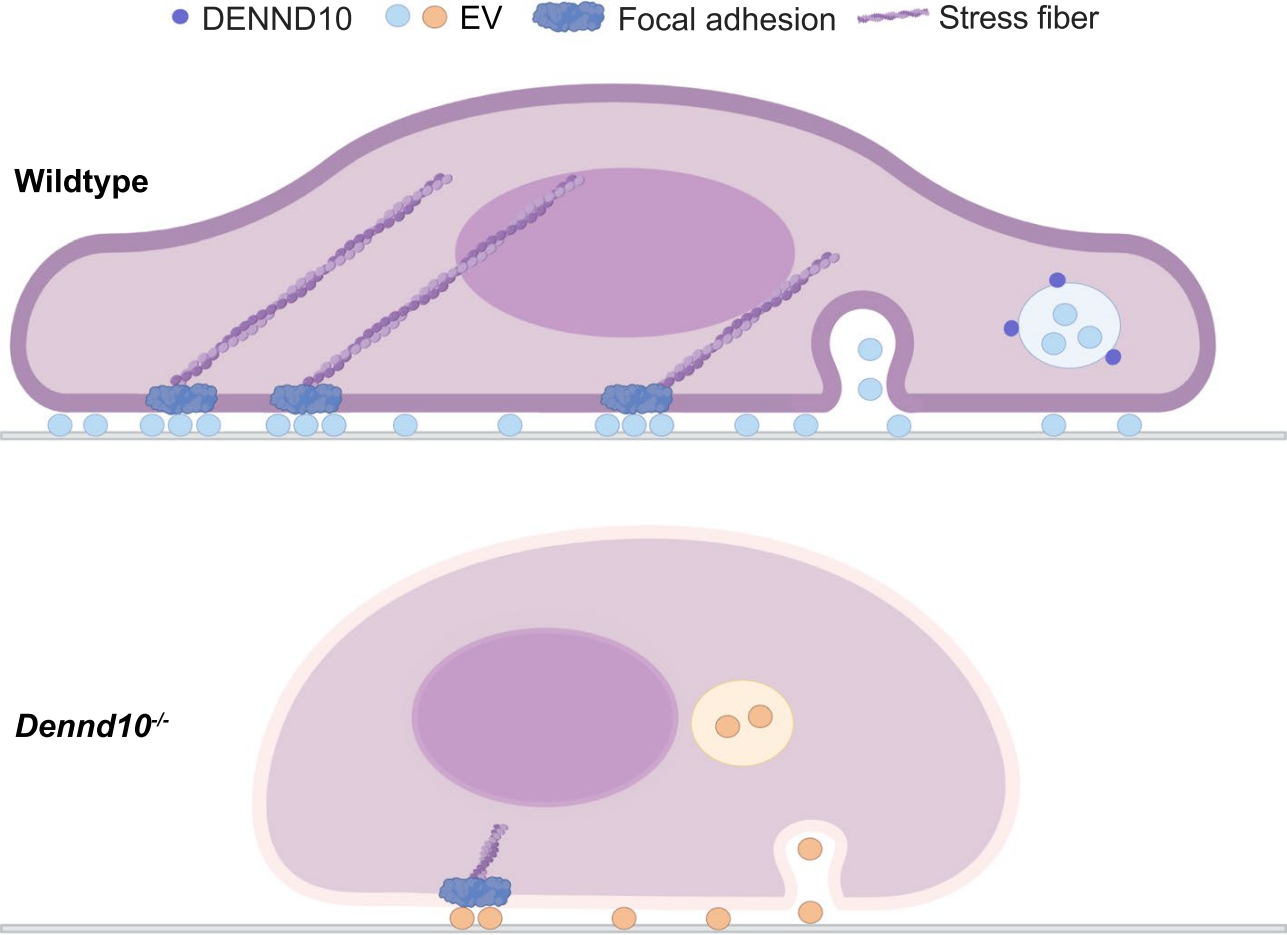
Diagram of DENND10 function in cancer cell migration via regulating EV release. DENND10 is mainly localized in multivesicular late endosomes and lysosomes, which are one primary source of EVs. EVs could serve as adhesion hotspots and provide spatial cues to facilitate cell migration by virtue of ECM and adhesion proteins displayed on their surface. DENND10 depletion results in decreased EV quantity and altered EV composition, especially in the profiles of ECM and cell adhesion molecules. Consequently, cell migration is impaired in DENND10-KO cells due to decreased formation of crucial cytoskeletal structures such as focal adhesions and stress fibers. Illustrated with BioRender (https://biorender.com/)

Taken together, our study showed that DENND10 regulates both the quantity and composition of autocrine tumor EVs, which contribute to the deposition of cargoes, particularly extracellular matrix proteins, within the tumor microenvironment, thereby facilitating efficient cell migration (Fig. 9).

## Discussion

In this study, we found that the endosomal protein DENND10 is involved in the migration of breast cancer cells by regulating the release of autocrine EVs. These findings could explain, at least in part, the adverse prognosis of breast cancer patients with high DENND10 expression. Our study underscores the importance of autocrine EV in tumor microenvironment and also reveals DENND10 as a potential target for metastatic cancer.

Our findings suggest that DENND10 modulates the migration and metastasis potential of breast cancer cells. Metastasis typically occurs late in disease progression. Consistent with this, when breast cancer patients were stratified based on ER, HER2, and KI67 expression (Additional file 1: Figure S1A), we noted that the impact of high DENND10 expression could be recognized at earlier time points (~ 25 months) in luminal B (characterized by high proliferation rates) and basal (HER2-/ER-) patients. In contrast, in the Luminal A cohorts, which are known for low proliferation and slower progression, this effect is observed much later (> 50 months). This suggests that DENND10's influence on disease progression may manifest differently depending on the subtype of breast cancer, with particular importance in highly aggressive cancers.

Altering the biogenesis of EVs could potentially remodel the tumor microenvironment. Studies have shown that tumor cells deliver PD-L1-containing EVs to suppress T cell activation, and downregulation of EV biogenesis induces systematic anti-tumor immune responses [46, 47]. Inhibiting EV production in melanoma [5] or breast cancer cells [48] decreases their metastatic potential. In addition, suppressing EV release in prostate cancer cells abolished the activation of tumor-promoting stroma [49]. These results suggest that targeting EV biogenesis is a promising anti-tumor strategy. Commonly used perturbations of EV biogenesis include depletion of the small GTPase Rab27 or inhibition of ceramide synthesis [46]. However, the contribution of other intracellular trafficking steps to EV bioactivities in the context of tumor development has been little explored.

In the current study, we have found that DENND10 regulates both the quantity and composition of EVs. This is in contrast to Rab27a knockdown, which reduces EV secretion but does not significantly affect the composition of EVs [50]. The uniqueness of DENND10 likely comes from its interaction with both small GTPases and the CCC-Retriever cargo-sorting complexes. DENND10 belongs to the DENN domain family, which generally acts as GEFs to activate Rab small GTPases. We had previously found that DENND10 interacts with Rab27a in a nucleotide-dependent manner [11]. Consistently, DENND10 knockout was phenotypically similar to Rab27a knockdown cells in multiple aspects, including accumulation of lysosomal proteins [49], reduction of EV release [50], and impaired migration [48]. Additionally, several recent structural studies [12–14] identified DENND10 as an integral subunit of the CCC-Retriever (Commander) endosomal complexes, a highly conserved protein machinery involved in endosomal sorting and recycling [51, 52]. We have confirmed this interaction ourselves (manuscript in preparation). Intriguingly, the depletion of COMMD5, another subunit of the CCC complex, resulted in disorganized cytoskeleton and impaired directional migration [53], similar to DENND10-KO phenotypes. The CCC-Retriever complexes are important for the recycling of many surface proteins, including multiple integrin and adhesion proteins [54]. Consistently, we observed that levels of integrin subtypes and adhesion proteins carried by EVs were affected by loss of DENND10 (Fig. 7F). Therefore, DENND10 could act through both Rab proteins and the CCC/Retriever complexes to modulate EV release and content. Future studies will reveal how DENND10 orchestrates its regulation on Rab activities and its role in the CCC-Retriever complex.

DENND10 deletion resulted in multiple changes in EV compositions, particularly remodeled ECM components and cell adhesion proteins. Multiple studies have identified ECMs and integrins on the surface of EVs [34, 35]. EV-associated integrin subtypes have been shown to determine the organ tropism of tumor metastasis [55]. EV-carried ECM molecules such as fibronectin could form cell adhesion hotspots and provide spatial cues to facilitate efficient cell migration [36–39]. In our case, selected collagen subtypes, as well as laminins, were mostly affected in DENND10-KO EVs. We further showed that exogenous application of ECMs rescued the morphology and migration of DENND10 cells, strongly suggesting that these defects mainly resulted from the changes in the quantity and ECM composition of EVs. These results could position DENND10 as a promising target for perturbating EV biogenesis to inhibit tumor metastasis.

Aside from ECM and adhesion proteins, other changes in DENND10-KO EVs included increased chromatin-binding proteins, decreased proteasome subunits, and reduced mitochondrial contents. It is conceivable that these changes would further alter the bioactivities of DENND10-KO EVs. Previous studies have shown increased proteasomes [8] and decreased nucleic acid-binding proteins [33] in EVs from metastatic cancer cells. EV-packaged mitochondria could influence the metabolic activity of target cells [56] or serve as a mechanism for the clearance of damaged mitochondria [57, 58]. How these EV cargoes may be involved in breast cancer metastasis still requires further research.

Finally, it should be noted that both pro-tumor immune suppression and anti-tumor immune responses could be elicited by tumor-derived EVs, depending on the context [59]. As EVs are inherently heterogeneous in nature, targeting EV biogenesis in cancer therapy should consider multiple factors, including clinical stages of cancer development, tissue origin of tumor types, and the mechanisms of intracellular biogenesis.

## Conclusions

In conclusion, this study unveiled novel physiological functions for the ancient protein DENND10, which plays a critical role in the coordination between the release of autocrine EVs and tumor cell migration. These results provided a new molecular target for the treatment of EV-dependent invasive tumors.

## Methods

### Cell Culture

4T1 cells were purchased from the Cell Bank of the Chinese Academy of Sciences (Shanghai, China, SCSP-5056) and cultured in RPMI 1640 medium (Invitrogen, Shanghai, China, C22400500BT) supplemented with 10% fetal bovine serum (Biological Industries, Kibbutz Beit Haemek, Israel, 04–001-1ACS), 1% GlutaMAX (Invitrogen, 35050061) and 1% Penicillin/Streptomycin solution (Hyclone, Logan, UT, USA, SV30082.01). Human MDA-MB-231 cells were a gift from Dr. Yufang Shi at Soochow University and cultured in DMEM/F12 medium supplemented as above. HEK293T cells were previously described [11] and cultured in DMEM medium (Invitrogen C11965500BT) supplemented as above. CRISPR knockout cell lines (NC and DENND10-KO 4T1 cells) and stable knockdown cell lines (shNC and shDENND10 MDA-MB-231 cells) were maintained in full medium containing 2 μg/mL puromycin.

### Antibodies

The following antibodies were obtained from Cell Signaling Technology (Beverly, MA, USA): LC3A/B (D3U4C) (#12741), total 4E-BP1 (53H11) (#9644), phospho-4E-BP1 (Thr37/46) (#2855), total p70-S6K (49D7) (#2708), phospho-p70-S6K (Thr389) (#9205), total mTOR (#2983), phospho-mTOR (Ser2448) (#1356), Alix (3A9) (#2171), Flotillin-1 (D2V7J) (#18,634), β-actin (13E5) (#4970) for immunofluorescence, α-tubulin (DM1A) (#3873) for immunofluorescence. Other antibodies used: LAMP1 (Santa Cruz Biotechnology, Santa Cruz, CA, USA, sc-19992), M6PR (Abcam, Cambridge, UK, ab32815) for immunofluorescence, M6PR (BBI Life Sciences, Shanghai, China, D154247) for western blotting, CD63 (BioLegend, San Diego, CA, USA, 143,902), Cathepsin D (Abcam, ab75852), Vinculin (PTM Biolabs, Hangzhou, China, PTM-5168), β-Actin (Ruiying Biological, Suzhou, China, RLT0099) for immunoblotting.

### Gene Knockout and Knockdown

A pair of CRISPR guide RNAs were designed to target the first ATG-containing exon of the mouse *Dennd10* gene: 5’-AAG TCC CGT CCC TTC CGG CA-3’ and 5’-CGG AGT CGG GCT GAT CGG TG-3’. They were cloned into the lentiCRISPR v2 vector [60] together and expressed under two separate U6 promoters from the same plasmid. Lentivirus packaging and infection were performed as previously described [11]. Infected cells were selected with 2 μg/mL puromycin. Single clones were obtained by serial dilution and then screened with qRT-PCR for the expression of *Dennd10*. Two independent clones (KO1 and KO5) were used for downstream analysis. 4T1 cells infected with non-targeting control lentiviral particles (NC) were used as the wild-type control.

Lentiviral mediated shRNA knockdown of human *DENND10* expression in MDA-MB-231 cells was performed with the same targeting sequences previously described in [11]: GGA GAG TTA TAT TGC AGT TCT (#1) and GCA ACA GAC CAG ACC TCT ATG (#2).

### Cell Proliferation

Cell proliferation was measured with the Cell Counting Kit-8 (CCK-8) assay (Dojindo, Kumamoto, Japan, CK04) according to the manufacturer’s manual. Briefly, cells were seeded in a 96-well plate at 2.5 × 10^3^ cells/well and settled for 12 h. At every 24-h interval, 10 µl CCK-8 solution was added to each well and incubated for 2.5 h. Absorbance was measured at 450 nm. Five technical repeats were performed for each time point.

### EV Isolation and Analysis

EVs were isolated with a modified ExtraPEG method [61]. Cells were seeded in 10-cm culture dishes at 8.5 × 10^5^ cells per dish and grew until 80% confluency before being changed into serum-free medium. After 24 h, the conditioned serum-free media were collected for exosome isolation. The media were centrifuged at 500 g for 5 min and then at 2, 300 g for 30 min at 4 °C to remove dead cells and debris. The supernatant was then mixed with an equal volume of 16% PEG (Beyotime, ST483)/1 M NaCl solution and incubated overnight at 4 °C. The mixture was centrifuged at 10, 000 g for 15–20 min at 4 °C. The pellet was resuspended in PBS buffer and saved in low-adsorption Eppendorf tubes. For western analysis in Fig. 1G, EVs derived from twelve dishes were concentrated in 200 µl PBS and equal volumes of EVs (15 µl, corresponding to ~ 7 × 10^6^ cells) were used for each lane. For rescuing cell morphology, migration, and invasion in Figs. 4 and 5, EVs were added to the medium at 10 µg/ml.

For particle concentration and size distribution, EV samples derived from three 10-cm plates were diluted 20–50 fold and probed with a NanoAnalyzer (NanoFCM, Xiamen, China, N30E). Note that vesicle size values measured from NanoFCM are generally smaller than those from nanoparticle tracking analysis (NTA) [62]. For TEM analysis, 10 µl EV sample was pipetted onto a copper grid, precipitated for 1 min, and blotted dry. The EVs were then negatively stained by 10 µl uranyl acetate for 1 min and blotted dry again. The grid was dried at room temperature for several minutes and imaged with an electron microscope (Hitachi, HT-7700) at 100 kV. Both particle and TEM analyses were performed by Oebiotech Inc. (Shanghai, China).

### Cell Spreading

For cell spreading, DENND10-KO or NC cells were seeded at 3 × 10^5^ cells/well in a six-well polystyrene plate (Nest Scientific, Wuxi, China, 703,001). Cell morphology was imaged with a 20 × objective on a Nikon Eclipse TS2 microscope at 12–24 h after plating. Three to five random fields were imaged for each sample. The percentage of fully spread cells was counted in each field manually by an experimenter blind to the sample label.

For rescue of cell spreading with ECM coating, culture dishes were pre-coated for 2 h at 37 °C with collagen I (Cellvis, 200,110–10, diluted in 6 mM acetic acid), laminin (Sigma, L2020, diluted in PBS), or fibronectin (SolarBio, F8180, diluted in PBS) at 15 µg/ml, or poly-L-lysine (Biosharp, BS099A) at 0.1 mg/ml, washed with PBS and used immediately.

### Transwell Migration and Invasion

4T1 or MDA-MB-231 cells were seeded in the Transwell chamber (Corning Inc, Corning, NY, USA, Cat No. 3422, pore size 8 µm) at 1 × 10^5^ cells per well in 200 µL serum-free medium. For invasion assay, Matrigel (Corning, 356,234) was diluted with serum-free medium to 0.3 mg/ml, applied to the upper chamber at 30 µg/well, and incubated for 3 h at 37 °C before use. For experiments with ECM rescue, the Transwell membrane was pre-coated on the underside by immersion in 15 µg/ml collagen I, laminin, or fibronectin solution for 2 h and used at 2.5 × 10^4^ cells per well. The chambers were placed in a 24-well plate (Nest, 702,001) filled with 750 µl of 10% FBS-containing medium per well. After incubation for 24 h, cells that migrated to the bottom side of the membrane insert were fixed with paraformaldehyde, stained with crystal violet, and imaged with a 10 × objective on an inverted TK-10 microscope (Tike-tec, Shanghai, China). The number of migrated cells per field was counted with ImageJ and normalized to the corresponding control.

### Wound Healing Assay

For scratch wound healing assay, NC or DENND10-KO 4T1 cells were seeded in a 6-well plate at 8 × 10^5^ cells per well to reach full confluency at 24 h. Scratches were made with a 10 µl pipette tip in the middle of the cell layer. The medium was washed out with PBS and replaced with serum-free medium to minimize the effect of proliferation. The scratches were imaged at 0 and 48 h when NC cells reached full wound closure. Quantitation of the wound area was done with the Wound Healing Size tool plugin [63] in ImageJ.

### Tumor metastasis

All animal experimental procedures were approved by the Animal Ethics Committee of Soochow University (Suzhou, Jiangsu, China). NC and DENND10-KO 4T1 cells were resuspended in PBS and injected into the fourth mammary gland of female BALB/c mice at 5 × 10^4^ cells per animal. The mice were kept in a pathogen-free animal facility under a 12-h light/12-h dark cycle with unlimited access to food and water. Tumor volume and body weight were monitored twice a week to avoid potential tumor overgrowth. After four weeks, the animals were euthanized by CO_2_ with a flow rate of 30% air displacement per minute. Euthanasia was confirmed by cervical dislocation. Macro-metastases to the lungs were manually counted.

### Fluorescence Microscopy

A total of 3 × 10^5^ cells were seeded onto coverslips in a 6-well plate. The next day, cells were fixed with 4% paraformaldehyde (Beyotime, P0099) and permeabilized with 0.1% Triton X-100. For immunofluorescence experiments, cells were blocked with 2% goat or donkey serum diluted in PBS, incubated with primary antibodies at 4 °C overnight, and then secondary antibodies for 1 h, stained with 0.1 µg/ml DAPI (Sigma, St Louis, MO, D9542), and mounted. The following secondary antibodies were used: Alexa Fluor 488 goat anti-rabbit IgG (Beyotime, A0423), Alexa Fluor 488 goat anti-mouse IgG (Beyotime, A0428), Alexa Fluor 555 donkey anti-mouse IgG (Beyotime, A0460), Cy3 goat anti-rat IgG (Beyotime, A0507). Slides were imaged on a Leica SP8 confocal microscope with a 63 × objective (N.A. = 1.4). For PMA stimulation, cells were pretreated with 1 µM Phorbol-12-Myristate-13-Acetate (PMA) (Santa Cruz, sc-3576A) for 30 min before fixation. For F-actin staining, permeabilized cells were incubated with 100 nM FITC-Phalloidin (Solarbio, Beijing, China, CA1620) diluted in 1% BSA for 30 min at room temperature before imaging. The percentage of perinuclear clustering of M6PR and LAMP1 was quantified using CellProfiler (v. 4.2.6) [64] as described in [65].

### Autophagy

4T1-NC and DENND10-KO cells were seeded at a density of 6 × 10^5^ cells per well in a six-well plate. After 24–36 h, autophagy was induced by incubating the cells in serum-free medium with or without Bafilomycin A1 (CST, 54645) for 8 h. Cells were then lysed and analyzed by Western blotting for β-actin and LC3A/B.

### Cathepsin B Activity Assay

To assess lysosomal proteolytic activities, NC and DENND10-KO 4T1 cells were seeded at a density of 8 × 10^4^ cells per well in a 24-well plate. After 24 h, the culture medium was replaced with 480 μl of fresh culture medium supplemented with 20 μl of 25 × Cathepsin B Magic Red (MR) substrates (Abcam, ab270772). The cells were then incubated at 37 °C for 30 min in the dark. Subsequently, cells were washed with PBS twice and fixed with 4% paraformaldehyde for 15 min. After another PBS wash, cells were mounted using SlowFade Diamond Antifade Mountant with DAPI (Invitrogen, S36968) and imaged immediately on a Leica SP8 confocal microscope with a 63 × objective (N.A. = 1.4). All slides were imaged with the same laser setting. Cathepsin B MR puncta were segmented and quantified using CellProfiler (v. 4.2.6) [64] by following the Speckle Counting pipeline (https://cellprofiler.org/examples).

### Cell Viability Assay

DENND10-KO or NC cells were seeded in 35 mm culture dishes (Nest, 706001) at 3 × 10^5^ cells per dish. After 24 h, cells were washed three times with PBS, and incubated for 20 min with phenol-red free DMEM-F12 (Gibco, 11039–021) supplemented with 10% FBS, 2 μM Calcein-AM, 4 μM EthD-I (Solarbio, CA1631) and 1 μg/ml Hoechst 33,342 (from Abcam, ab270772). Live cells were imaged on a Leica SP8 confocal microscope with a 10 × objective. To examine cell viability under the condition of scratch wound healing assay, cells were seeded at 8 × 10^5^ cells per dish to reach full confluency at 24 h. The medium was then replaced with serum-free medium. After another 48 h, cell viability was examined as above.

### Western Blotting

For the preparation of whole-cell lysates, cells were lysed in the lysis buffer (50 mM Tris pH 7.5, 150 mM NaCl, 0.5% Triton X-100, and proteinase inhibitor cocktails) by rocking for 30 min and spun down at 10, 000 g for 10 min at 4 °C. For the preparation of EV proteins, EV samples were lysed by mixing with Laemmli sample buffer (without reducing reagents), boiled at 95 °C, and spun down briefly. Protein concentrations were determined by BCA assay (Pierce, Rockford, USA, 23,227). Samples were separated on SDS-PAGE electrophoresis, transferred to PVDF membrane, blocked with 5% nonfat milk in 1 × TBS-T buffer, and incubated with appropriate primary antibodies overnight at 4 °C, followed by incubation with corresponding secondary antibodies for one hour: HRP conjugated goat anti-rabbit IgG (H + L) (Proteintech, SA00001-2), HRP conjugated goat anti-rat IgG (Proteintech, SA00001-15), or HRP linked horse anti-mouse IgG (Cell Signaling Technology, #7076). The blot was washed in 1 × TBS-T buffer and developed with ECL substrates (Millipore, Bedford, MA, USA, WBKLS0500).

### qRT-PCR

Total RNA was extracted from 4T1 cells using TriZol (Sangon, Shanghai, China, B511311). Reverse transcription was carried out with PrimeScript RT Master Mix (Takara, RR036A). Quantitative PCR (qPCR) was set up with SYBR Premix EX Taq II (Takara, Otsu, Japan, RR820A) and run on an ABI 7500 Real-Time PCR system. The following primers were used. DENND10_F: 5’-GTT GTG GCA ATG GAC ACC CA-3’, DENND10_R: 5’-GGC TGT CGT GGA AGG ATA ACA-3’; TFEB_F: 5’-AGC AGG TGG TGA AGC AAG AG-3’, TFEB_R: 5’-CAG GTG ATG GAA CGG AGA C-3’. Expression of RPL32 was used as the internal control: L32_F: 5’-TTA AGC GAA ACT GGC GGA AAC-3’, and L32_R: 5’-TTG TTG CTC CCA TAA CCG ATG-3’. Relative gene expression was determined with the 2^−∆∆Ct^ method.

### Mass Spectrometry

Proteomics experiments were performed at the PTM Biolab Inc (Hangzhou, China). EV samples were lysed by sonication in the presence of 1% protease inhibitor cocktail. BCA assay was used to determine protein concentration. Equal masses (60 µg) of proteins were reduced by 5 mM DTT at 56 °C for 30 min and alkylated with 11 mM iodoacetamide for 15 min in the dark. Proteins were digested by trypsin (1:50, m/m) overnight. The resultant peptides were separated on a NanoElute UHPLC system, ionized, and analyzed with a timsTOF Pro instrument (Bruker Daltonik, Germany). The following parameters were used: scan range 100–1700 m/z; data collection mode at PASEF; dynamic exclusion at 30 s. MS/MS spectra were searched against the mouse proteome database (17,089 entries, July 2021) with MaxQuant (v. 1.6.15.0). False discovery rate (FDR) for peptide matching was set at 1%.

A total of 29,069 peptides were obtained, including 27,284 unique peptides, which were mapped to 3323 identified proteins. Among these proteins, 2518 proteins were present in both NC and DENND10-KO EVs and deemed as “quantifiable”. Protein abundance in each sample was calculated by LFQ intensity. For each protein, all LFQ intensities were scaled to the mean of all samples. Fold change (FC) was calculated by the ratio between the mean of DENND10-KO samples and NC samples. For screening of DEPs, protein abundance values were log-transformed and then compared by Student’s t-test. Statistical tests were not performed on proteins with fewer than two measured values in the control or knockout group. Raw p-values were adjusted for multiple comparisons using the Benjamini & Hochberg procedure to obtain FDR values.

### Bioinformatics and Data Analysis

The relationship between *DENND10* expression and cancer patient survival was assessed using KM plotter (kmplot.com). Patients with breast cancer or gastric cancer were split into two groups based on the median of DENND10/FAM45A expression (Affy ID: 221804_s_at) in all patients under study. In addition, the breast cancer patients were further stratified using the St Gallen classification scheme as described in [16] (Basal: ER-/HER2-; luminal A: ER + /HER2-/KI67 low; luminal B: ER + /HER2-/KI67 high or ER + /HER2 + ; HER2 + : HER2 + /ER-). All other parameters were set as default. Kaplan–Meier survival plots were drawn to compare differences in disease progression between the two groups. Hazard ratio (HR) with 95% confidence interval and logrank p-values were calculated using Cox regression. The GSE46563 dataset was downloaded from the GEO database (https://www.ncbi.nlm.nih.gov/geo/) and analyzed by survival analysis.

EV proteins identified in the proteomics study were compared to the Exocarta (Ver. 5, July 29, 2015) and Vesiclepedia (Ver. 4.1, August 15, 2018) databases. Human gene IDs in the database were mapped to homologous mouse gene IDs with Metascape [66]. Functional enrichment analysis was performed with the FunRich (Ver. 3.1.4) software [67], a tool recommended by the EV research community. The STRING database (Ver. 11.0, https://string-db.org/) was used to retrieve associations among DEPs. Interactions with a confidence score > 0.7 were visualized with Cytoscape (Ver. 3.9). Volcano plot and heatmaps were generated using ggplot2 and pheatmap in R.

### Statistics

Statistical analysis was performed in Prism (GraphPad, v 9.0) with testing methods indicated in the figure legends. Results were shown as mean ± SEM. A value of *p* < 0.05 was considered statistically significant.

### Supplementary Information

**Additional file 1:** **Figure S1**. DENND10 expression is correlated with poor patient survival in gastric cancer and breast cancer. **Figure S2**. DENND10 deletion does not affect cell proliferation. **Figure S3**. The effect of DENND10 deletion on the endolysosome system in breast cancer cells. **Figure S4**. DENND10 deletion does not affect cell viability. **Figure S5**. DENND10 is important for the migration of breast cancer cells. **Figure S6**. Loss of DENND10 does not reduce total levels of actin. **Figure S7**. Loss of DENND10 results in cytoskeleton reorganization. **Figure S8**. Major functional clusters in EV proteins that are differentially expressed in DENND10-KO cells.

**Additional file 2: Table S1**. List of DENND10 expression in human breast cancer cell lines with adherent growth. **Table S2**. List of all proteins identified in the mass spectrometry analysis of EVs from 4T1-NC and DENND10-KO cells. **Table S3**. List of selected proteins of interest in the 4T1 EV mass spectrometry dataset. **Table S4**. Overlap among 4T1-EV proteomics dataset in this study, Vesiclepedia, and Exocarta databases. **Table S5**. Pathway enrichment of cellular components for all identified proteins in the 4T1-EV proteomics dataset. **Table S6**. List of all DEPs in DENND10-KO EVs. **Table S7**. Pathway enrichment of cellular components for DEPs. **Table S8**. Protein-protein interaction network among DEPs.

**Additional file 3:** Individual data point values in the study.

**Additional file 4:** Uncropped western blots used in the study.

## Data Availability

All data generated or analysed during this study are included in this published article, its supplementary information files and publicly available repositories. The individual data point values of the replicates are listed in Additional file 3. The following public datasets were analyzed: KM plot (https://kmplot.com/analysis/index.php), CCLE (https://depmap.org/portal/gene/DENND10?tab=characterization), and GSE644563 (https://www.ncbi.nlm.nih.gov/geo/query/acc.cgi?acc=GSE46563). The mass spectrometry proteomics data contained in the manuscript have been deposited to the ProteomeXchange Consortium with the dataset identifier PXD038264 (http://www.ebi.ac.uk/pride/archive/projects/PXD038264).

## Abbreviations

EV: Extracellular vesicles
DENND10-KO: DENND10 knockout
NC: Cells infected with non-targeting control viruses
MVE: Multivesicular endosomes
DENN: Differentially expressed in normal cells and neoplasia
GEF: Guanine nucleotide exchange factors
ECM: Extracellular matrix
PKC: Protein kinase C
F-actin: Filamentous actin
PMA: Phorbol-12-Myristate-13-Acetate
TEM: Transmission electron microscopy
nFCM: Nano flow cytometry
MR: Magic Red
PCA: Principal component analysis
DEP: Differentially expressed protein
FDR: False discovery rate
FC: Fold change
