# Supplementary material for: Endosomal protein DENND10/FAM45A integrates extracellular vesicle release with cancer cell migration
Source: BMC Biol. 2024 Jul 10;22:154. doi: 10.1186/s12915-024-01948-4 (PMC11234546; doi:10.1186/s12915-024-01948-4)
Supplement: Supplementary file 1 — Additional file 1: Figure S1. DENND10 expression is correlated with poor patient survival in gastric cancer and breast cancer. Figure S2. DENND10 deletion does not affect cell proliferation. Figure S3. The effect of DENND10 deletion on the endolysosome system in breast cancer cells. Figure S4. DENND10 deletion does not affect cell viability. Figure S5. DENND10 is important for the migration of breast cancer cells. Figure S6. Loss of DENND10 does not reduce total levels of actin. Figure S7. Loss of DENND10 results in cytoskeleton reorganization. Figure S8. Major functional clusters in EV proteins that are differentially expressed in DENND10-KO cells. [file 12915_2024_1948_MOESM1_ESM.docx]

## Endosomal protein DENND10/FAM45A integrates extracellular vesicle release with cancer cell migration

**Shenqing Sun^1#^, Qian Li^1#^, Ganggang Liu^1#^, Xiaoheng Huang^1^, Aiqing Li^1^, Haoran Guo^1^, Lijuan Qi^1^, Jie Zhang^1^, Jianrui Song^2*^, Xiong Su^1,3,4*^, Yanling Zhang^1,3,4*^**

^1^School of Life Sciences, Suzhou Medical College of Soochow University, Suzhou, 215123, China.

^2^Wisdom Lake Academy of Pharmacy, Jiangsu Provincial Higher Education Key Laboratory of Cell Therapy Nanoformulation, Xi’an Jiaotong-Liverpool University, Suzhou 215123, China

^3^MOE Key Laboratory of Geriatric Diseases and Immunology, Suzhou Medical College of Soochow University, Suzhou, 215123, China.

^4^Suzhou Key Laboratory of Systems Biomedicine, Suzhou Medical College of Soochow University, Suzhou, 215123, China.

^#^ Contributed equally.

* To whom correspondence should be addressed:

Yanling Zhang; School of Life Sciences, MOE Key Laboratory of Geriatric Diseases and Immunology, Suzhou Medical College of Soochow University, Suzhou, 215123, China; Email: [yanlzhan@suda.edu.cn](mailto:yanlzhan@suda.edu.cn); [Tel: 0086-512-65880108](Tel:0086-512-65880108); ORCID: 0000-0002-1483-9021

Xiong Su; Department of Biochemistry and Molecular Biology, MOE Key Laboratory of Geriatric Diseases and Immunology, Suzhou Key Laboratory of Systems Biomedicine, Suzhou Medical College of Soochow University, Suzhou, 215123, China; Email: [xsu@suda.edu.cn](mailto:xsu@suda.edu.cn); [Tel: 0086-512-65883622](Tel:0086-512-65883622); ORCID: 0000-0001-8998-1826

Jianrui Song; Wisdom Lake Academy of Pharmacy, Xi’an Jiaotong-Liverpool University, Suzhou 215123, China; Email: [jianrui.song@xjtlu.edu.cn](mailto:jianrui.song@xjtlu.edu.cn); Tel: 0086-512-88161358; ORCID: 0000-0001-5472-4483

Running title: DENND10 modulates autocrine EVs


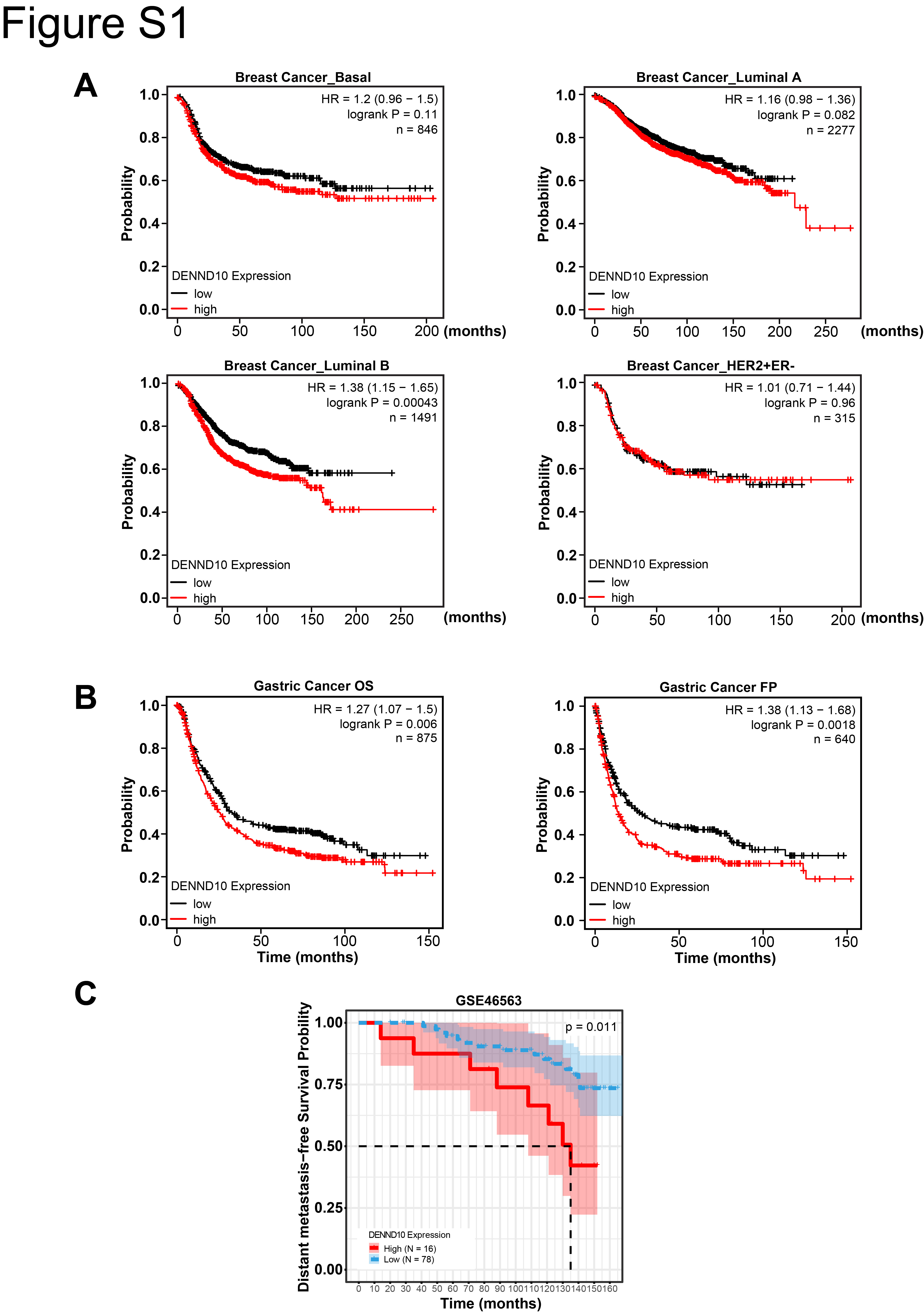


**Figure S1. DENND10 expression is correlated with poor patient survival in gastric cancer and breast cancer.**

**A.** The effect of DENND10 expression in breast cancer subtypes. Breast cancer patients in Figure 1A (N = 4, 929) were stratified into four groups using the St Gallen scheme: 1) Basal: ER-/HER2-; 2) luminal A: ER+/HER2-/KI67 low; 3) luminal B: ER+/HER2-/KI67 high or ER+/HER2+; 4) HER2+: HER2+/ER-. The number of patients in each group is indicated in each panel. HR, hazard ratio. Data source: KM Plotter.

**B.** Overall survival (OS) and time to first-progression (FP) in patients with gastric cancer. Patients were divided into high expression (red) and low expression (black) based on the median expression of DENND10. HR, hazard ratio. Data source: KM Plotter.

**C.** Distant metastasis-free survival in breast cancer patients from the GSE46563 dataset. Patients were divided into high expression (red) and low expression (black) based on an automatically selected cutoff that resulted in the largest discrepancy between the two groups.


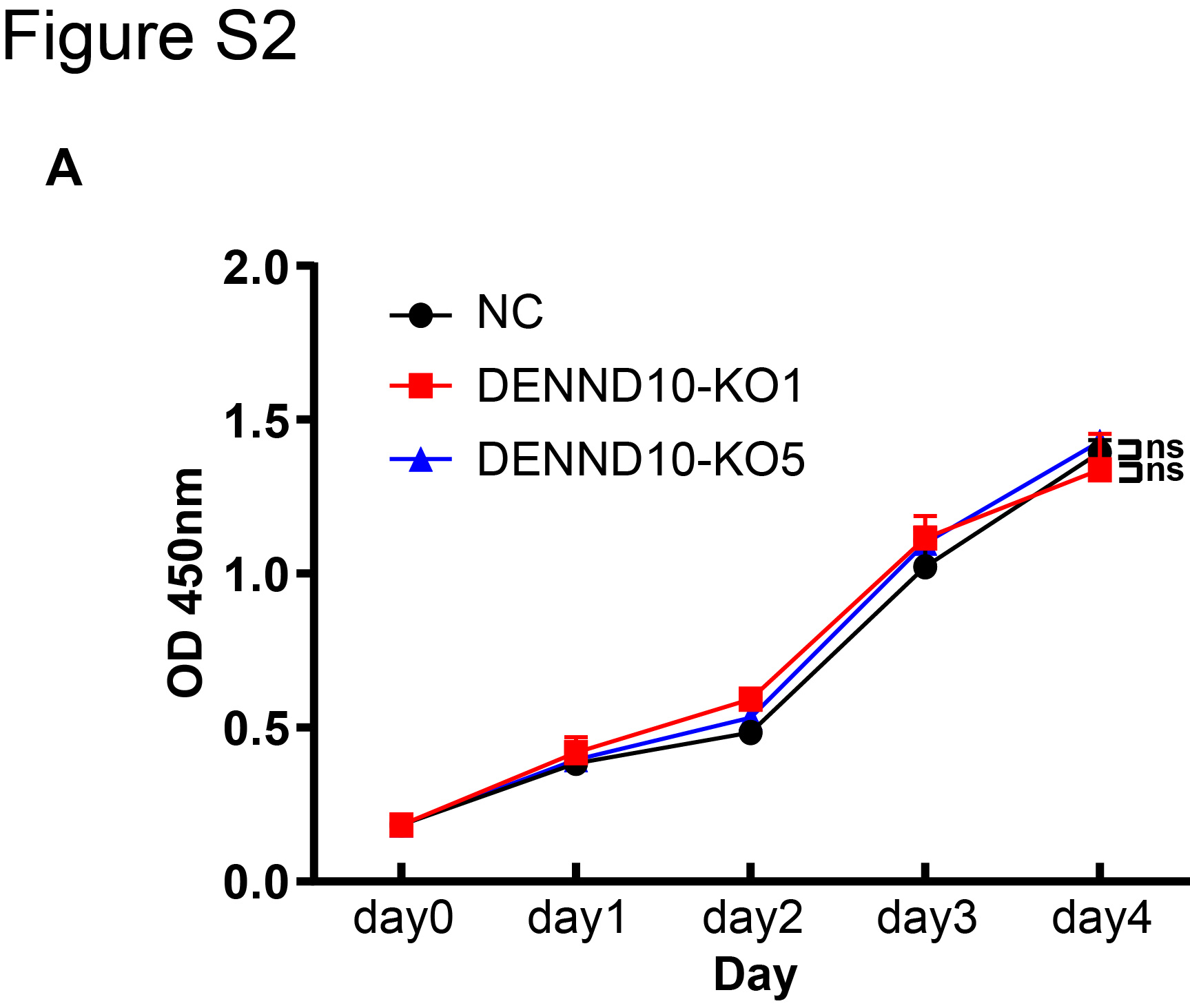


**Figure S2. DENND10 deletion does not affect cell proliferation.**

On days 0-4 after plating, cell proliferation was measured by CCK-8 for NC and DENND10-KO cells. Error bars, SEM (N = 3, see Additional file 3). Statistical significance was determined by Student’s t-test between DENND10 knockout cells and NC cells. ns, non-significant.


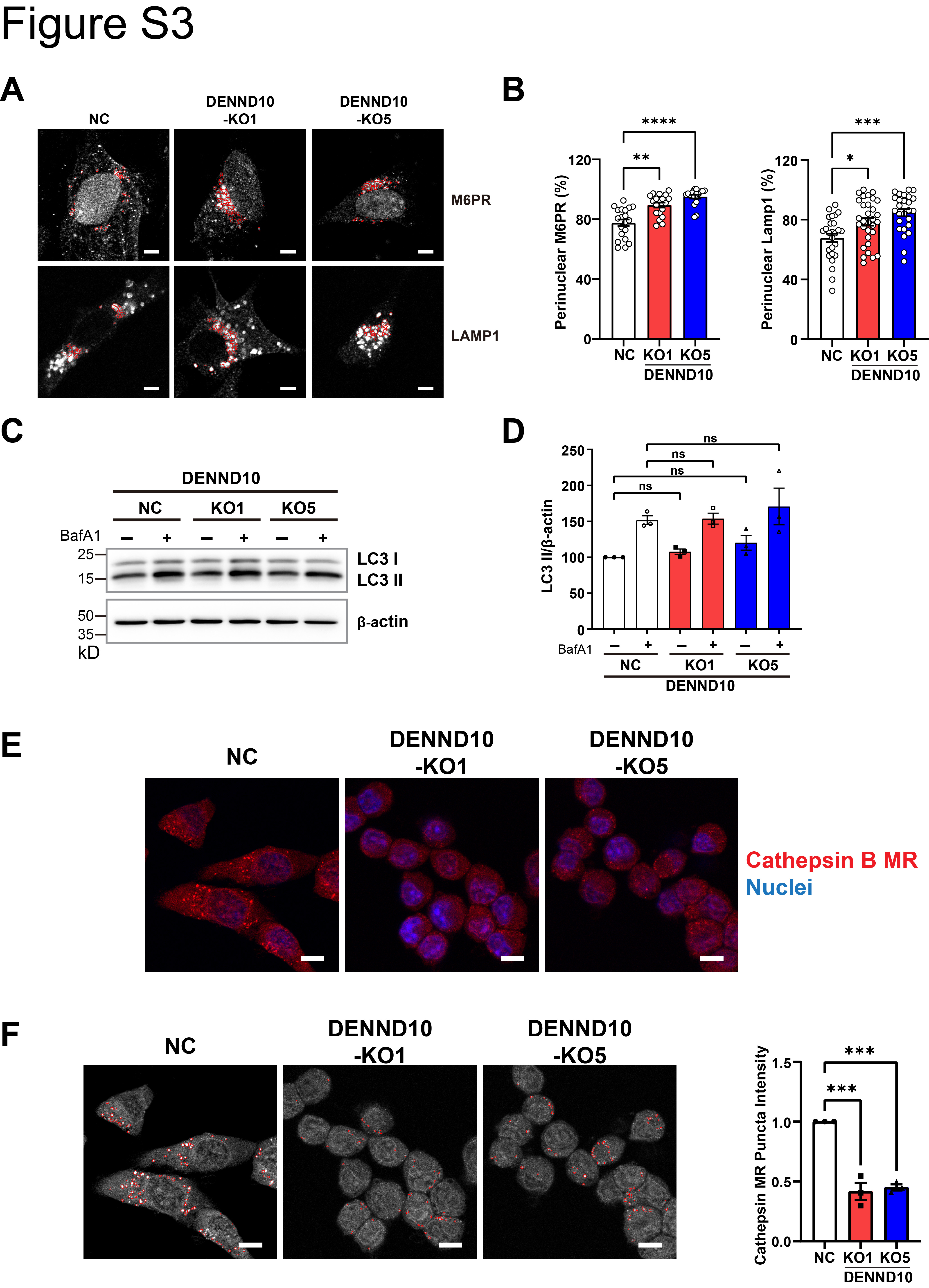


**Figure S3. The effect of DENND10 deletion on the endolysosome system in breast cancer cells.**

**A-B.** M6PR and LAMP1 are clustered in the perinuclear region in DENND10-KO cells.

**A.** Examples of perinuclear M6PR and LAMP1 puncta (red outlined) segmented by CellProfiler. The perinuclear region was defined as 35 pixels (3.2 μm) surrounding the nucleus. Scale bars: 5 μm.

**B.** Quantitation of perinuclear M6PR and LAMP1 signals as a percentage of total signal intensity in the whole cell. Error bars, SEM. N = 18-20 cells for M6PR and 26-31 cells for LAMP1 from four independent experiments. Significance was determined with the Kruskal-Wallis test, followed by Dunn’s multiple-comparison test. *, p < 0.05; **, p < 0.01; ***, p < 0.001; ****, p < 0.0001.

**C-D.** Autophagy levels do not change significantly in DENND10-KO cells.

**C.** Levels of LC3 proteins in NC and DENND10-KO cells in the presence or absence of Bafilomycin A1 (BafA1), an inhibitor of lysosomal proteases.

**D.** Quantitation of LC3-II intensity normalized to the actin loading control. Error bars, SEM. N = 3 independent experiments (See Additional file 3). Significance was determined with one-way ANOVA, followed by Holm-Šídák’s multiple-comparison test. ns, non-significant.

**E-F.** Proteolytic activities within individual lysosomes are decreased in DENND10-KO cells.

**E.** NC and DENND10-KO cells were incubated with the Cathepsin B Magic Red (MR) substrates for 30 min and fixed. Upon cleavage within lysosomes, MR substrates release the cresyl violet fluorophores (red), which are trapped in acidic organelles. All cells were imaged with the same laser illumination setting. Scale bars: 10 μm.

**F.** Quantitation of Cathepsin B MR puncta intensity. Left: MR puncta recognized by the CellProfiler were outlined in red. The nucleus region was excluded to avoid nonspecific staining. Right: The average of MR puncta intensities was normalized to the NC cells. Error bars, SEM. N = 3 independent experiments with 50-100 cells in each genotype group (See Additional file 3). Significance was determined with One-way ANOVA followed by Dunnett's multiple comparisons test. ***, p < 0.001.


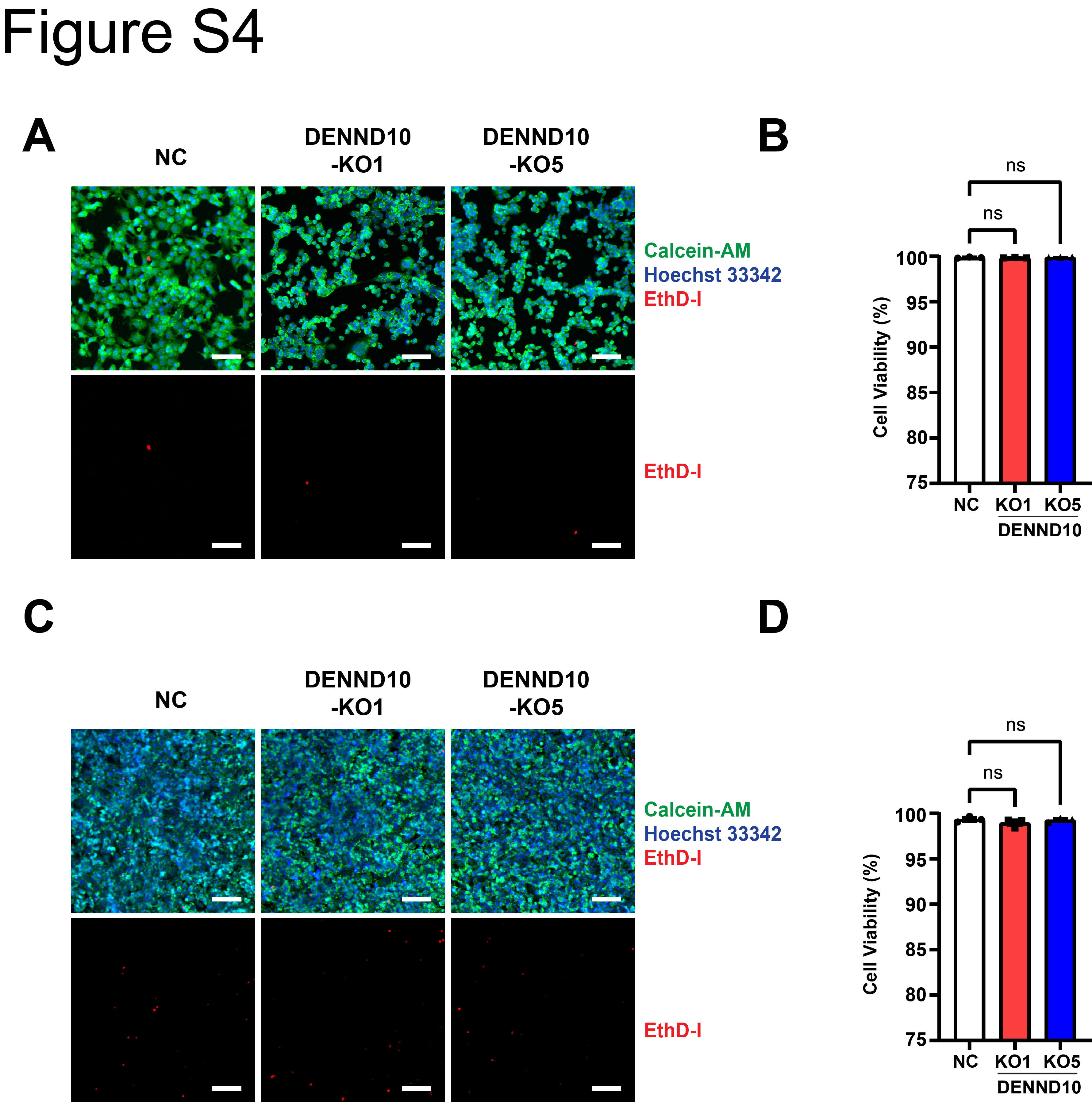


**Figure S4. DENND10 deletion does not affect cell viability.**

**A.** NC and DENND10-KO cells were seeded at 3 × 10^5^ cells per 35-mm dish. Cell viability was examined using Calcein-AM (green fluorescence in live cells), EthD-I (red fluorescence in dead cells), and Hoechst 33342 (blue fluorescence staining the nuclei).

**B.** Quantitation of the percentage of viable cells in (**A**) calculated by (1 – the percentage of red nuclei in total nuclei) × 100. Error bars, SEM (N = 3, see Additional file 3). Significance was determined with One-way ANOVA followed by Dunnett's multiple comparisons test. ns, non-significant.

**C.** NC and DENND10-KO cells were seeded at 8 × 10^5^ cells per 35-mm dish. After 24 hours, cells were cultured in serum-free medium for 48 hours before being assessed for cell viability.

**D.** Quantitation of the percentage of viable cells in (**C**). Error bars, SEM (N = 3, see Additional file 3). ns, non-significant.


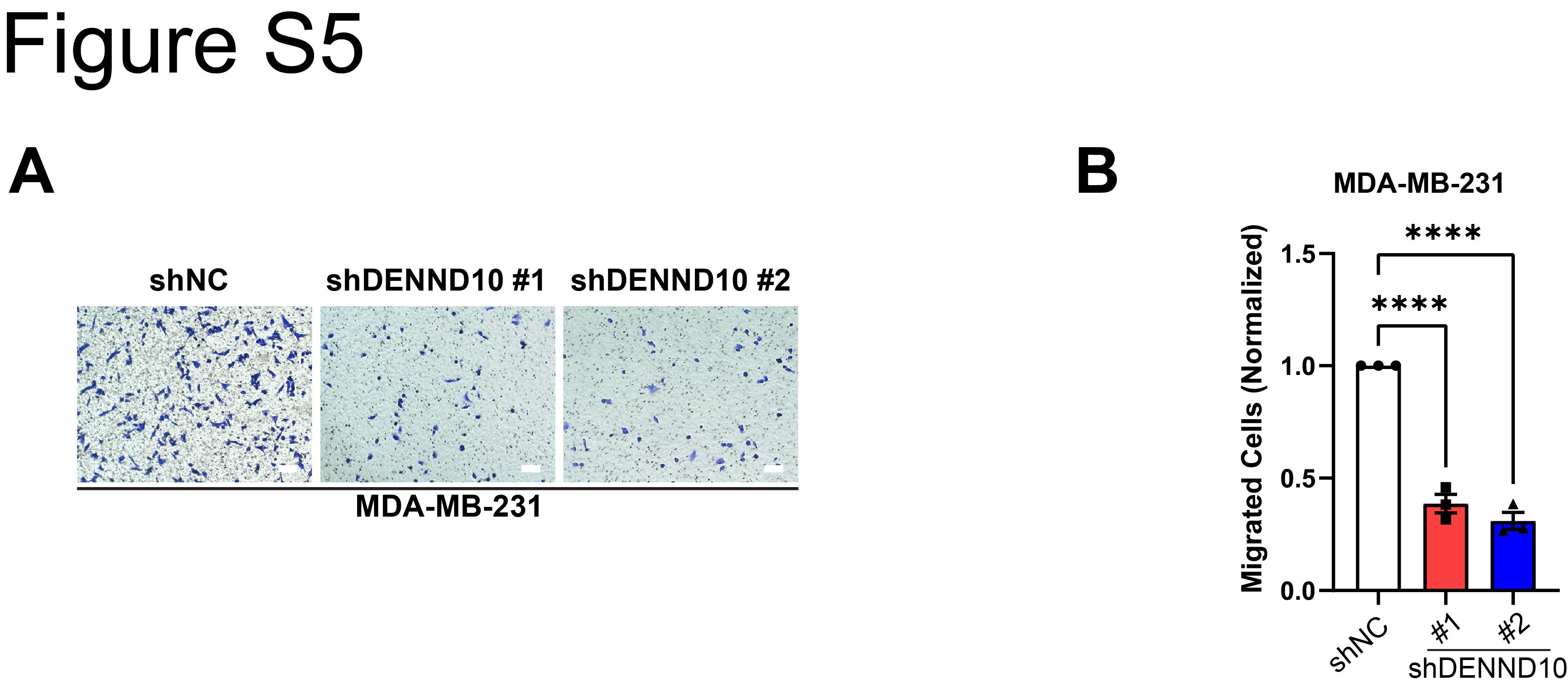


**Figure S5. DENND10 is important for the migration of breast cancer cells.**

**A.** DENND10 inhibition led to defective migration across the transwell membrane. Human MDA-MB-231 cells were stably transfected with two independent shRNAs against *DENND10* gene (shDENND10 #1 and #2) or non-targeting control shRNA (shNC). Scale bars: 100 μm.

**B.** Quantitation of migrated cells in (**A**). The number of migrated cells per field was normalized to the control. Error bars, SEM (N = 3, see Additional file 3). Significance was determined by one-way ANOVA followed by Dunnett’s multiple comparisons test. ****, p < 0.0001.


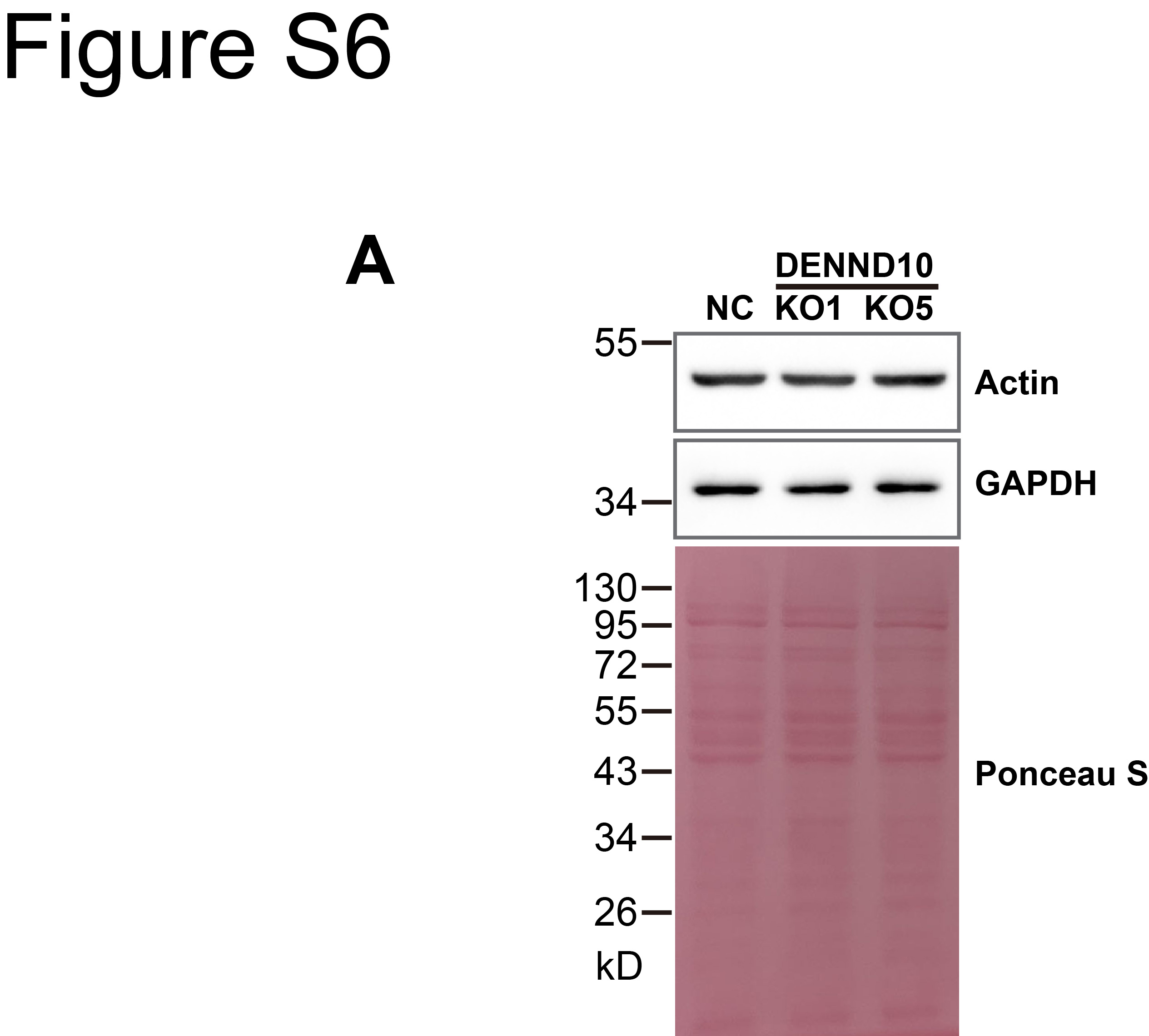


**Figure S6. Loss of DENND10 does not reduce total levels of actin.**

**A**. Expression levels of actin and GAPDH were assessed in NC and DENND10-KO cells, together with the corresponding Ponceau S staining showing overall protein loadings.


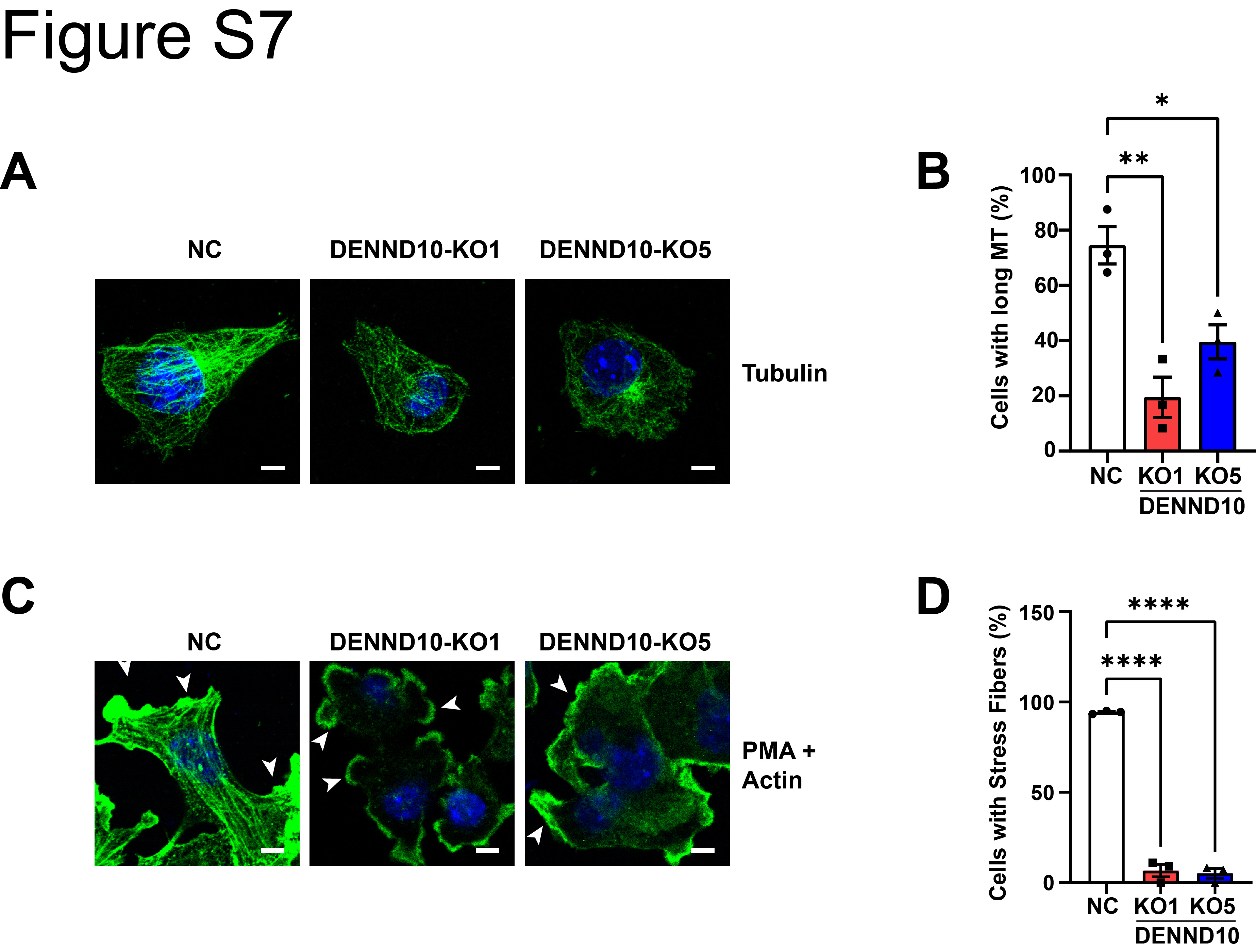


**Figure S7. Loss of DENND10 results in cytoskeleton reorganization.**

**A**. NC and DENND10-KO cells were cultured for 24 hours before fixation and stained with anti-tubulin. Anti-tubulin staining exhibited a long-range organized array of microtubules in NC cells, but appeared disorganized in DENND10-KO cells.

**B**. Quantitation of the percentage of cells with long-range microtubule (MT) structures in (**A**). Error bars, SEM (N = 3, see Additional file 3).

**C.** Cells were treated with 1 μM PMA for 30 minutes to induce membrane ruffles (arrowheads) before fixation.

**D.** Quantitation of the percentage of cells with stress fibers in (**C**). Error bars, SEM (N = 3, see Additional file 3).

Statistical significance in (**B**) and (**D**) was analyzed by one-way ANOVA followed by Dunnett’s multiple comparisons test. *, p < 0.05; **, p < 0.01; ****, p < 0.0001.


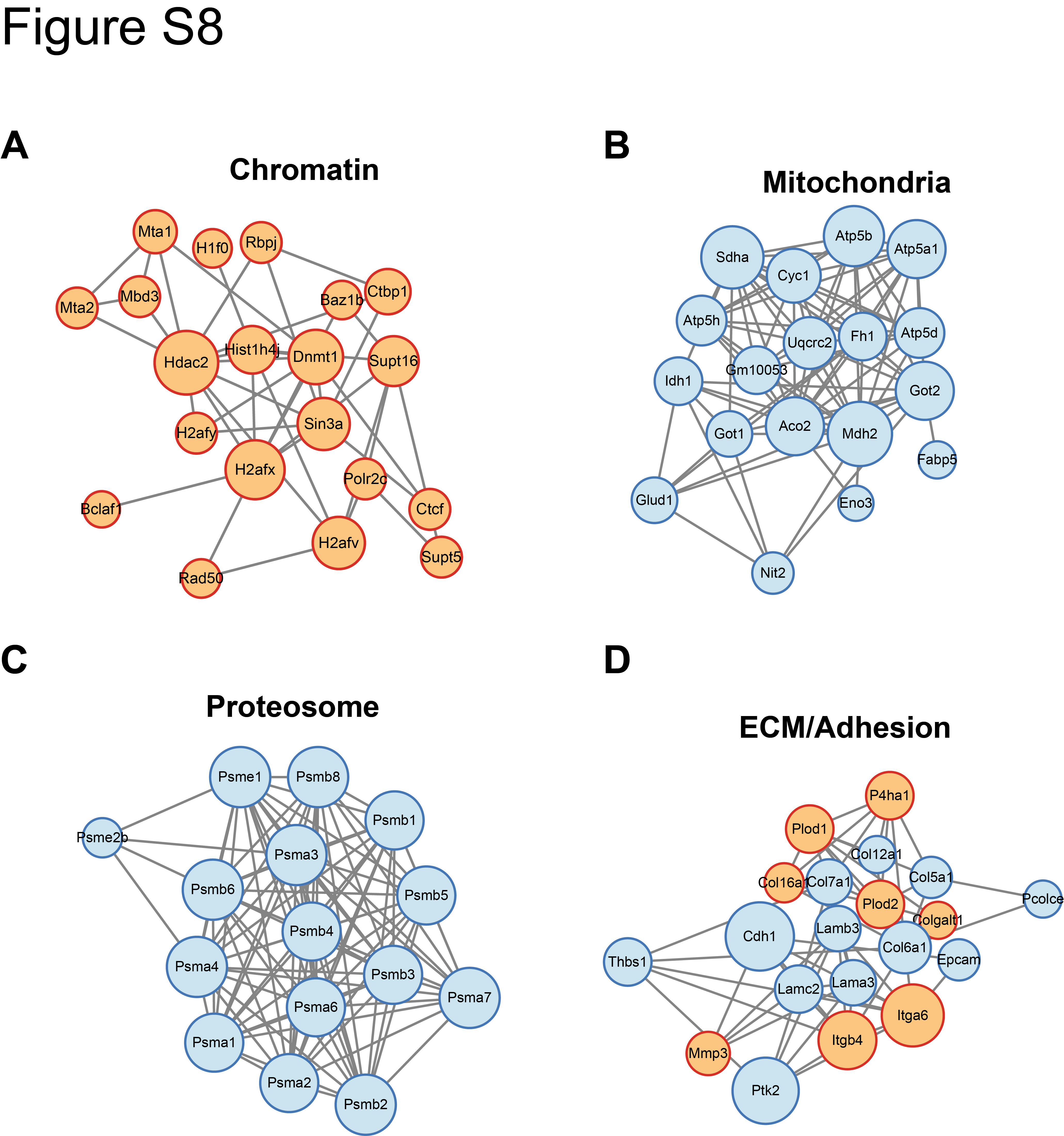


**Figure S8. Major functional clusters in EV proteins that are differentially expressed in DENND10-KO cells.**

**A.** Chromatin-binding proteins were up-regulated in DENND10-KO EVs.

**B.** Mitochondrial proteins were down-regulated in DENND10-KO EVs.

**C.** Proteasomal proteins were down-regulated in DENND10-KO EVs.

**D.** ECM and adhesion proteins exhibited distinct subtype profiles in DENND10-KO EVs.

Up-regulated DEPs were colored light red, and down-regulated DEPs were colored light blue.
