# Supplementary material for: Endosomal protein DENND10/FAM45A integrates extracellular vesicle release with cancer cell migration
Source: BMC Biol. 2024 Jul 10;22:154. doi: 10.1186/s12915-024-01948-4 (PMC11234546; doi:10.1186/s12915-024-01948-4)
Supplement: Supplementary file 2 — Additional file 2: Table S1. List of DENND10 expression in human breast cancer cell lines with adherent growth. Table S2. List of all proteins identified in the mass spectrometry analysis of EVs from 4T1-NC and DENND10-KO cells. Table S3. List of selected proteins of interest in the 4T1 EV mass spectrometry dataset. Table S4. Overlap among 4T1-EV proteomics dataset in this study, Vesiclepedia, and Exocarta databases. Table S5. Pathway enrichment of cellular components for all identified proteins in the 4T1-EV proteomics dataset. Table S6. List of all DEPs in DENND10-KO EVs. Table S7. Pathway enrichment of cellular components for DEPs. Table S8. Protein-protein interaction network among DEPs. [file 12915_2024_1948_MOESM2_ESM.zip › Supplemental table legends.docx]

## Endosomal protein DENND10/FAM45A integrates extracellular vesicle release with cancer cell migration

**Shenqing Sun^1#^, Qian Li^1#^, Ganggang Liu^1#^, Xiaoheng Huang^1^, Aiqing Li^1^, Haoran Guo^1^, Lijuan Qi^1^, Jie Zhang^1^, Jianrui Song^2*^, Xiong Su^1,3,4*^, Yanling Zhang^1,3*^**

^1^Department of Biochemistry and Molecular Biology, Suzhou Medical College of Soochow University, Suzhou, 215123, China.

^2^Wisdom Lake Academy of Pharmacy, Xi’an Jiaotong-Liverpool University, Suzhou 215123, China

^3^MOE Key Laboratory of Geriatric Diseases and Immunology, Suzhou Medical College of Soochow University, Suzhou, 215123, China.

^4^Suzhou Key Laboratory of Systems Biomedicine, Suzhou Medical College of Soochow University, Suzhou, 215123, China.

^#^ Contributed equally.

* To whom correspondence should be addressed:

Yanling Zhang; Department of Biochemistry and Molecular Biology, MOE Key Laboratory of Geriatric Diseases and Immunology, Suzhou Medical College of Soochow University, Suzhou, 215123, China; Email: [yanlzhan@suda.edu.cn](mailto:yanlzhan@suda.edu.cn); [Tel: 0086-512-65880108](Tel:0086-512-65880108); ORCID: 0000-0002-1483-9021

Xiong Su; Department of Biochemistry and Molecular Biology, MOE Key Laboratory of Geriatric Diseases and Immunology, Suzhou Key Laboratory of Systems Biomedicine, Suzhou Medical College of Soochow University, Suzhou, 215123, China; Email: [xsu@suda.edu.cn](mailto:xsu@suda.edu.cn); [Tel: 0086-512-65883622](Tel:0086-512-65883622); ORCID: 0000-0001-8998-1826

Jianrui Song; Wisdom Lake Academy of Pharmacy, Xi’an Jiaotong-Liverpool University, Suzhou 215123, China; Email: [jianrui.song@xjtlu.edu.cn](mailto:jianrui.song@xjtlu.edu.cn); Tel: 0086-512-88161358; ORCID: 0000-0001-5472-4483

Running title: DENND10 modulates autocrine EVs

## Supporting information

**Supplemental Table Legends**

**Table S1. List of DENND10 expression in human breast cancer cell lines with adherent growth.** Data source: CCLE database (https://depmap.org/portal/download/).

**Table S2. List of all proteins identified in the mass spectrometry analysis of EVs from 4T1-NC and DENND10-KO cells.** Original LFQ values, normalized quantitation values, p-values, and FDR values were calculated as described in the methods.

**Table S3. List of selected proteins of interest in the 4T1 EV mass spectrometry dataset.** Several categories of proteins were included in this table: DENND10 itself, common EV markers, cell adhesion/ECM proteins, proteasome subunits, metabolic enzymes, and chromatin-binding proteins. Up-regulated DEPs were highlighted in red, and down-regulated DEPs were highlighted in green. Some proteins did not have p-values because of the absence in one group of samples (i.e., DENND10).

**Table S4. Overlap among 4T1-EV proteomics dataset in this study, Vesiclepedia, and Exocarta databases.** Uniprot entries used in mass spectrometry were mapped to Entrez gene IDs with Metascape.

**Table S5. Pathway enrichment of cellular components for all identified proteins in the 4T1-EV proteomics dataset.** The background used was the FunRich (human only) database included in FunRich (v3.1.4). Proteins in 4T1-EVs were mapped to corresponding human IDs with Metascape.

**Table S6. List of all DEPs in DENND10-KO EVs.**

**Table S7. Pathway enrichment of cellular components for DEPs.**

Enrichment p-values were calculated with FunRich (v3.1.4). The background used was the Uniprot database.

**Table S8. Protein-protein interaction network among DEPs**.

Interactions among proteins were derived from the STRING database. Edges with confidence > 0.7 were included.
