# Supplementary figures and images for: Endosomal protein DENND10/FAM45A integrates extracellular vesicle release with cancer cell migration
Source: BMC Biol. 2024 Jul 10;22:154. doi: 10.1186/s12915-024-01948-4 (PMC11234546; doi:10.1186/s12915-024-01948-4)

# Figure 1G

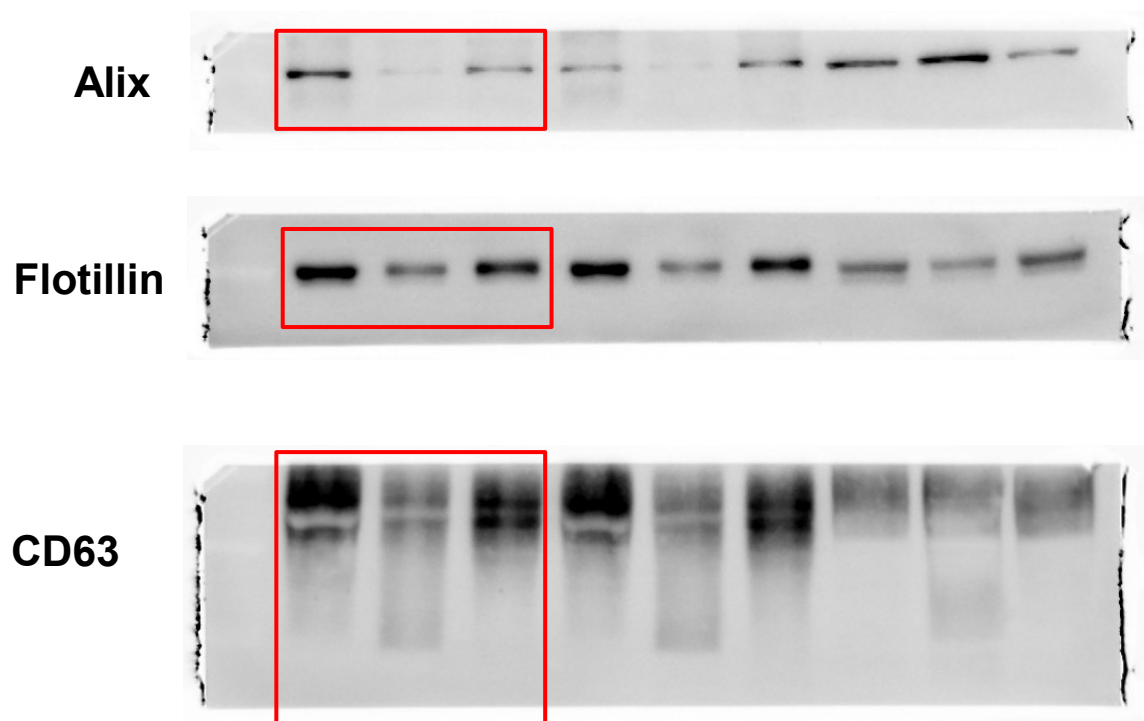

# Figure 2C

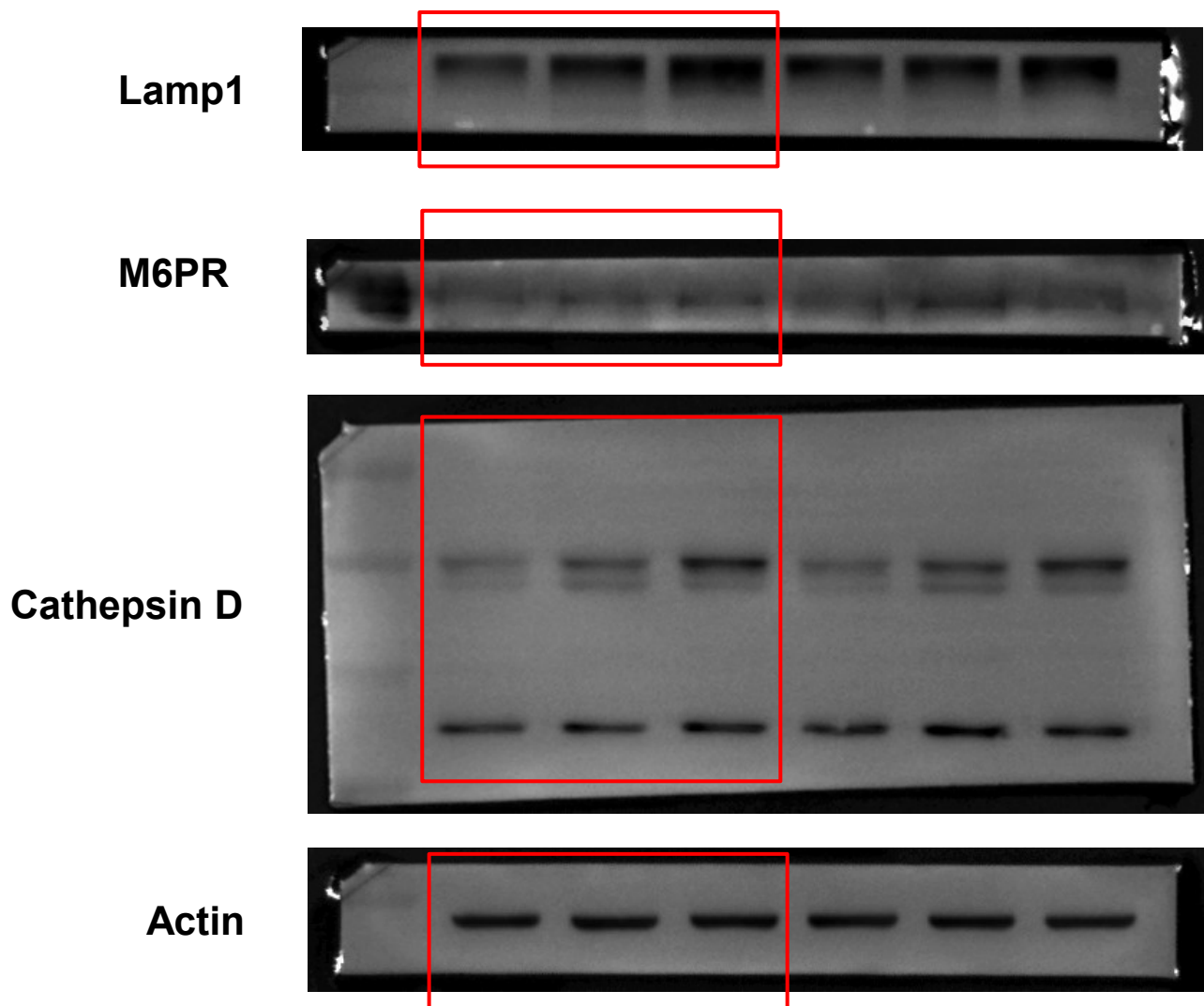

# Figure 2G

t-mTOR

p-mTOR

t-p70

p-p70

t-4EBP1

p-4EBP1

Actin

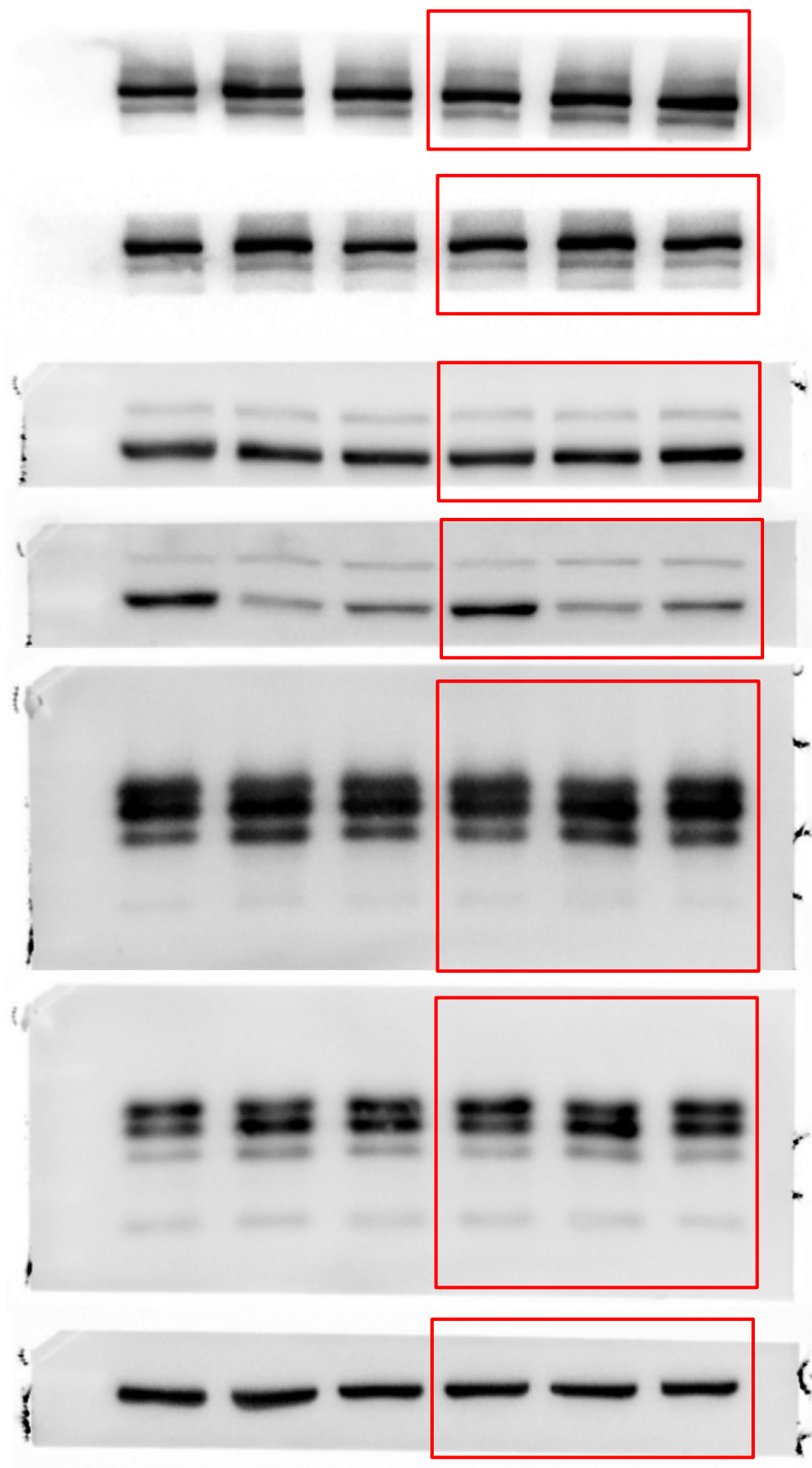

# Figure S3C

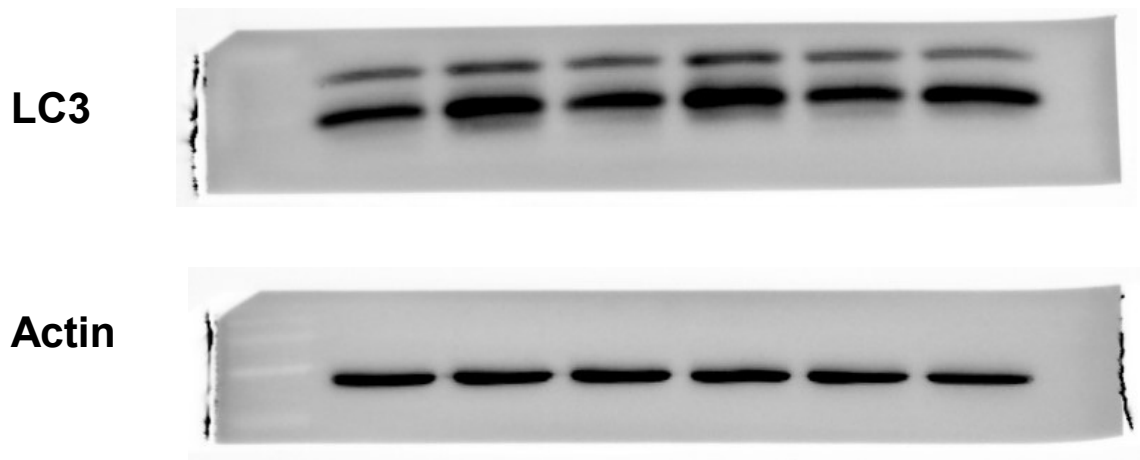

# Figure S6

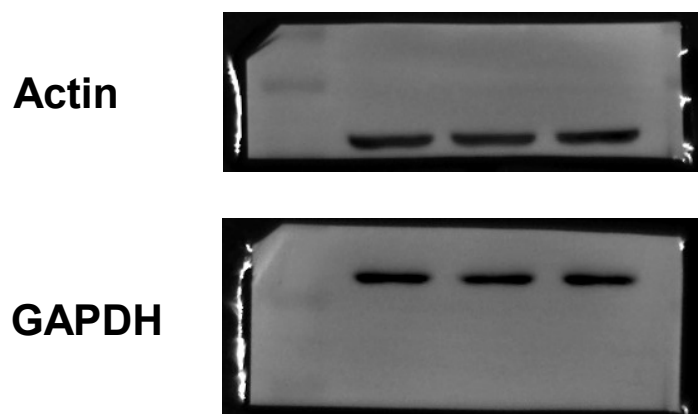

Supplement: Supplementary file 4 — Additional file 4: Uncropped western blots used in the study. [file 12915_2024_1948_MOESM4_ESM.pdf]
